# Characterization of a Gene Family Encoding SEA (Sea-urchin Sperm Protein, Enterokinase and Agrin)-Domain Proteins with Lectin-Like and Heme-Binding Properties from *Schistosoma japonicum*

**DOI:** 10.1371/journal.pntd.0002644

**Published:** 2014-01-09

**Authors:** Evaristus Chibunna Mbanefo, Mihoko Kikuchi, Nguyen Tien Huy, Mohammed Nasir Shuaibu, Mahamoud Sama Cherif, Chuanxin Yu, Masahiro Wakao, Yasuo Suda, Kenji Hirayama

**Affiliations:** 1 Department of Immunogenetics, Institute of Tropical Medicine (NEKKEN) and Global COE Program, Nagasaki University, Sakamoto, Japan; 2 Department of Parasitology and Entomology, Faculty of Bioscience, Nnamdi Azikiwe University, Awka, Nigeria; 3 Laboratory on Technology for Parasitic Disease Prevention and Control, Jiangsu Institute of Parasitic Diseases, Meiyuan, Wuxi, People's Republic of China; 4 Department of Chemistry, Biotechnology and Chemical Engineering, Graduate School of Science and Engineering, Kagoshima University, Kohrimoto, Kagoshima, Japan; 5 SUDx-Biotec Corporation, Shiroyama, Kagoshima, Japan; University of Melbourne, Australia

## Abstract

**Background:**

We previously identified a novel gene family dispersed in the genome of *Schistosoma japonicum* by retrotransposon-mediated gene duplication mechanism. Although many transcripts were identified, no homolog was readily identifiable from sequence information.

**Methodology/Principal Findings:**

Here, we utilized structural homology modeling and biochemical methods to identify remote homologs, and characterized the gene products as SEA (**s**ea-urchin sperm protein, **e**nterokinase and **a**grin)-domain containing proteins. A common extracellular domain in this family was structurally similar to SEA-domain. SEA-domain is primarily a structural domain, known to assist or regulate binding to glycans. Recombinant proteins from three members of this gene family specifically interacted with glycosaminoglycans with high affinity, with potential implication in ligand acquisition and immune evasion. Similar approach was used to identify a heme-binding site on the SEA-domain. The heme-binding mode showed heme molecule inserted into a hydrophobic pocket, with heme iron putatively coordinated to two histidine axial ligands. Heme-binding properties were confirmed using biochemical assays and UV-visible absorption spectroscopy, which showed high affinity heme-binding (*K*_D_ = 1.605×10^−6^ M) and cognate spectroscopic attributes of hexa-coordinated heme iron. The native proteins were oligomers, antigenic, and are localized on adult worm teguments and gastrodermis; major host-parasite interfaces and site for heme detoxification and acquisition.

**Conclusions:**

The results suggest potential role, at least in the nucleation step of heme crystallization (hemozoin formation), and as receptors for heme uptake. Survival strategies exploited by parasites, including heme homeostasis mechanism in hemoparasites, are paramount for successful parasitism. Thus, assessing prospects for application in disease intervention is warranted.

## Author Summary

While isolating membrane-bound and secreted proteins as targets for *Schistosoma japonicum* vaccine, we identified a novel potentially functional gene family which had originated by a gene duplication mechanism. Here, we integrated structural homology modeling and biochemical methods to show that this gene family encodes proteins with sea-urchin sperm protein, enterokinase and agrin (SEA) –domain, with heme-binding properties. Typical of SEA-structural domains, the characterized proteins specifically interacted with glycosaminoglycans (GAGs), with implication in ligand gathering and immune-evasion. Consistent with modeled heme-binding pocket, we observed high affinity heme-binding and spectroscopic attributes of hexa-coordinated heme iron. Localization of the native gene-products on adult worm tegument and gastrodermis, host interfaces for heme-sequestration and acquisition, suggests potential roles for this gene family in heme-detoxification and heme-iron uptake.

## Introduction

Schistosomiasis still ranks as the most important helminthic infection; second only to malaria in its socioeconomic burden in the resource constrained tropics and subtropics. It affects over 200 million people worldwide with more than 700 million people at risk of getting infected [1]. Although an effective treatment is available (praziquantel), the fact that reinfection occurs very rapidly after mass treatment renders chemotherapy alone inadequate for disease control. It is opined that a prophylactic alternative applied singly or in combination with other interventions, even with limited efficacy in limiting transmission is the optimum approach [2]. This intervention is especially needed in *S. japonicum* endemic areas, where non-human mammalian hosts are complicating control efforts.

Schistosomes inhabit host vasculature, where they ingest erythrocytes and catabolize the host hemoglobin as a source of amino acids for their growth, development and reproduction [3]. However, large quantities of potentially toxic heme (Fe-protoporphyrin IX) are released as ‘byproducts’ of hemoglobinolysis [3]–[6]. The parasite is thus faced with the challenge of maintaining heme homeostasis by evolving strategies to sequester and detoxify heme [3], [5]–[9], and at the same time maintaining a heme acquisition mechanism to harness the needed iron from the heme molecules [4], [10]. Indeed, effective mechanisms for detoxification of toxic heme and controlled acquisition of heme iron are paramount for parasite survival and establishment. Such mechanisms are major targets of effective drugs against hemoparasites, including malaria and schistosomiasis [11]–[13]. However, information on the exact mechanisms and molecules involved in this ‘weak link’ is either lacking or equivocal [3]. Such molecular targets should be localized at the host-parasite interfaces in contact with the host erythrocytes.

The tegument and gastrodermis are syncytial layers lining the entire parasite surface and the parasite gut, respectively [14]–[16]. Heme liberated during hemoglobinolysis is sequestered in the parasite gastrodermis lining the gut lumen [4], [17], and subsequently detoxified to non-toxic crystalline aggregates called hemozoin [8], [9], [17], [18] and regurgitated. The exact mechanism is not fully understood but it is thought that heme-binding proteins initiate the nucleation step of the crystallization, while lipids mediate the elongation step in an amphiphilic interface created by lipid droplets in the gastrodermis and gut lumen [17], [19]. Equally, schistosomes like other obligate parasites scavenge molecules from the host, including heme as the major source of iron needed for development and reproduction [4], [10]. Also, newly penetrated schistosomulae obtain iron via heme-binding proteins on their teguments before their guts are developed [20]. Thus, heme-binding proteins that are localized at these interfaces are most likely involved in the parasite heme acquisition and detoxification.

Over the years, enormous resources and technologies have been channeled towards identifying molecular targets involved in several biological mechanisms utilized by parasites for effective parasitism. The recently completed genome [21], transcriptome sequences [22] and proteomic studies [23] of this parasite represent invaluable feats towards identifying such targets. Although the functions of many sequenced genes are readily known or inferred from their amino acid sequences, many of the genes that are potential determinants of successful parasitism sometimes do not have readily identifiable sequence homologs. This is a major challenge for placing the vast amount of *‘omics’* data into functional contexts for identifying genes of interest [24], [25]. As a matter of fact, several of such proteins presently annotated as ‘hypothetical proteins’ may well represent the missing link to filling the gene ‘gaps’ in our understanding of host-parasite interactions. Indeed, over 30% of *S. japonicum* proteins are yet of unknown functions [21]. Therefore, adopting novel strategies for the characterization of otherwise ‘hypothetical proteins’ is highly needed and can provide valuable functional clues that may not be readily identifiable from sequence data alone [24], [25].

Our group had utilized a signal sequence trap (SST) to isolate secreted and membrane binding antigens from *S. japonicum* with appreciable success [26]. Among the SST isolated candidates, we identified a novel gene family which we found to have originated through a repetitive element mediated DNA-level gene duplication mechanism [27]. Although several transcripts from ∼27 duplicons were identified, no sequence homolog was readily identifiable in other organisms. We here utilized an integrated strategy combining comparative structural homology modeling and biochemical analyses to identify remote structural homologs, and characterize an extracellular domain in this family as SEA (**s**ea urchin sperm protein, **e**nterokinase and **a**grin)-domain. Similar approach was used to further identify and characterize a functional heme-binding site on the SEA-domain. SEA-module is an extracellular structural domain originally identified in **s**ea urchin **s**perm protein, **e**nterokinase and **a**grin, the basis for the nomenclature [28]–[30]. The domain is found in several functionally diverse proteins, and is known to assist or regulate binding to carbohydrate moieties. SEA-domain evolved from the ancestral ferredoxin-like fold, which is able to acquire various active sites including heme-binding sites [24]. The identification of a functional heme-binding protein in this hemophagous trematode is a significant contribution to our understanding of the host-parasite interaction as regards heme homeostasis. The biological significance of this finding and the potential role of this gene family in parasitism are discussed in terms of the parasite biology and prospects for application in disease intervention.

## Materials and Methods

### Ethics statement

This study adhered strictly to the recommendations in the Fundamental Guidelines for Proper Conduct of Animal Experiment and Related Activities in Academic Research Institutions under the jurisdiction of the Ministry of Education, Culture, Sports, Science and Technology, Japan (Notice No: 71). All animal experiments were approved by Nagasaki University Board of Animal Research, according to Japanese guidelines for use of experimental animals (Approval No: 0809050699).

### Experimental animals

Six to eight weeks old Female BALB/c mice were purchased from SLC Inc. Labs, Japan. The CLAWN strain miniature pigs were from Japan Farm, Kagoshima, Japan. The miniature pigs were infected percutaneously with 200 *S. japonicum* cercariae.

### Molecular structure modeling and ligand-binding characterization

Multiple alignments were performed using NCBI BLAST and *Multialin* Interface [31]. Post translational modifications were predicted using YingOYang 1.2 [32]. Molecular structure modeling was performed by fold recognition and *ab-initio* structure prediction methods using Protein Homology/Analogy Recognition Engine (*Phyre* v2.0) [33] and *Rosetta* Full Chain Protein Structure Prediction Server [34]. Ligand binding analysis to identify potential ligands and their binding sites in the folded protein was performed using *3DLigandSite* server [35]. The modeled structures were analyzed using Discovery Suite 3.5 Molecular Visualizer, while the modeled receptor-ligand interactions were analyzed on the PyMol Molecular Graphics System, Version 1.6 (Schrodinger, LLC).

### Total RNA isolation, cDNA synthesis and quantitative real-time PCR

Total mRNA was purified from parasite egg, sporocyst, cercaria and schistosomula using Micro-to-Midi total RNA purification system (Invitrogen, USA), and from adult worms using NucleoSpin RNA II kit (Macherey-Nagel, Germany). Reverse transcription and amplification of the double stranded cDNAs were performed using Ovation Pico WTA System v2 (NuGEN, USA). For each candidate gene and the reference gene (*S. japonicum* β-Actin), PCR fragment was first cloned into pCR2.1 cloning vector and the resulting constructs used as templates for qPCR standards and for estimation of copy numbers. Relative expression of candidate genes in different developmental stages of the parasite was quantified using SYBR Premix *Ex Taq* II Reagents (Takara, Japan). Real-time PCR and data analysis were performed on AB 7500 Real-Time PCR Systems v2.0.5.

### Cloning, expression and purification of recombinant protein

The complete coding sequences of the candidates were amplified and cloned into the TOPO TA cloning site of the expression vector pcDNA4/HisMax and expressed in BL21 *E. coli* cells, and FreeStyle 293 expression system (Invitrogen, USA) for binding assays. We took advantage of His6 tag to purify the recombinant proteins using TALON Metal Affinity Resins (Clontech, USA). Purified proteins were concentrated and imidazole elution buffer exchanged using Amicon Ultra Centrifugal Filters (Millipore, USA). Size exclusion gel filtration was performed using Sephadex G-50 medium (GE healthcare, USA). For heme-binding assays, purified proteins from FreeStyle 293 cells were treated with enterokinase to remove tags and purified with EK-Away resin (Invitrogen, USA).

### Preparation of specific immune serum and monoclonal antibodies

Polyclonal mouse sera were produced against recombinant antigens by subcutaneous immunization of mice with 25 µg of purified recombinant proteins in 50 µl PBS, mixed with an equal volume of Gerbu Adjuvant 100 (GERBU Biotechnik, Denmark), on days 0, 21 and 42. Two weeks after the last inoculation, mice were exsanguinated to collect sera and spleens were aseptically obtained for monoclonal antibody preparation using the Clonacell-HY system (Stemcell Technologies, USA), according to manufacturer's instructions. The monoclonal antibodies were biotinylated using the one-step antibody biotinylation kit (Mitenylbiotech, USA).

### Immunolocalization

Freshly perfused adult *S. japonicum* were washed three times in PBS (pH 7.4) and fixed in 4% neutral paraformaldehyde at 4°C until use. The samples were alcohol dehydrated, embedded in paraffin, cut into 5–7 µm thin sections and then mounted on microscope glass slides. Paraffin sections were deparaffinized by incubating for 10 min in two changes of xylene and rehydrated by sequential 10 min incubations in 100%, 95%, 70% and 50% ethanol, before rinsing in two changes of double deionized water. Schistosomulae were prepared by mechanical transformation and washed in Hanks solution. After washing with distilled water, the juvenile worms were fixed in cold acetone for 2 hours. Two drops of acetone fixed schistosomulae were added to poly-L-lysine coated glass slides and dried overnight. Immunoperoxidase technique was then performed as in adult worm sections.

Immunoperoxidase staining and immunofluorescence assays were performed using minor modifications to the method detailed by [36]. Briefly, the sections for immunoperoxidase staining were treated with 3% H_2_O_2_ in PBS for 30 min to destroy endogenous peroxidase. All sections were blocked for non-specific binding with 5% skim milk in PBS for 1 h, and then incubated for 2 h at room temperature with biotinylated monoclonal antibody or immune sera as indicated in each case. After washing three times in PBS pH 7.4 for 5 min each, the sections were incubated in FITC conjugated secondary antibody for immune sera IFA. For biotinylated mAB IFA and immunoperoxidase assays, sections were incubated for 30 mins with streptavidin-FITC (1∶500) and streptavidin-HRP (1∶500) solution respectively. The immunoperoxidase sections were washed in PBS and treated with diaminobenzidine tetrahydrochloride (DAB) chromogen, according to manufacturer's instructions (Dako, Japan). After counterstaining immunoperoxidase sections with Mayer hematoxylin, all the sections were washed, dehydrated by passage through alcohol and xylene, mounted, and viewed under Keyence All-in-one Fluorescence Microscope (Keyence, USA). Pre-immune serum was used as negative control.

### Glycoprotein detection

For glycoprotein detection assay, SDS-PAGE fractionated purified recombinant proteins were stained using the Pierce Glycoprotein Staining Kit (Thermo Scientific, USA).

### Glycan binding analysis using Surface Plasmon Resonance (SPR)

We utilized array type sugar chip (SUDx-Biotec, Japan); which is an array of 48 structurally defined sugar chains (glycans) immobilized on a thin gold chip to analyze the interactions of the SEA-domain proteins with glycans using SPR imaging [37]. The surface plasmon is excited when light is focused on the opposite side of the chip. The reflective light is measurable and is altered in response to binding of the proteins to the immobilized glycans. This alteration of the surface plasmon (expressed as resonance units, RU) is directly proportional to change in bound mass of analytes. Real time measuring of the SPR RU was used to monitor changes in the surface concentration or amount of bound analytes (protein). One of the benefits of this SPR system is that the weak interactions, which are easily washed out in the regular array technology and therefore not recognized, can also be monitored in real time. We used this method to detect real-time biological interactions between several glycans and the characterized SEA-domain proteins. For assessing the specificity and affinity of the protein-glycan interactions, we used chondroitin sulfate GAG chip to measure the association and dissociation kinetics in real time to determine *K*_D_ of the binding_._

### Hemin-agarose binding assay

Hemin-agarose binding assay was applied to study heme binding as detailed by [38]. Briefly, 200 µl of hemin-agarose (Sigma-Aldrich, USA) was washed three times in 1 ml of 100 mM NaCl-25 mM Tris-HCl (pH 7.4) with centrifugation done at 750×*g* for 5 min. Hemin-agarose was incubated with protein (20 µg) for 1 h at 37°C with gentle mixing. After 4 washes to remove unbound proteins, the beads were incubated for 2 min with elution solution (2% (wt/vol) SDS and 1% (vol/vol) β-mercaptoethanol in 500 mM Tris HCl, pH 6.8), boiled at 100°C for 5 min; centrifuged, and the supernatant analyzed by SDS-PAGE.

### Heme peroxidase activity based heme-binding assay

Binding assay based on the peroxidase activity of bound heme was performed as detailed by [38], [39]. Briefly, micro-titer plate coated with serial dilutions of the recombinant protein was incubated with hemin (20 µg/100 µl) at 37°C for 1 h. The unbound hemin was removed and the wells were washed three times with PBS (pH 7.3). 50 µl of ready-to-use substrate tetramethylbenzidine/H_2_O_2__(_TMB) (Bangalore-Genei, India) was added and the reaction stopped after 15 min with addition of equal volume of 1N H_2_SO_4_. The OD_450_ was determined in an ELISA plate reader (Bio-Rad, USA). The amount of hemin bound to protein was calculated from a linear graph of the peroxidase activities of known concentrations of hemin.

### Heme spectrometric titration

Optical absorption spectrometric studies were performed on Hitachi U-3900H spectrophotometer according to method detailed by [40]. Briefly, the binding of proteins to heme was titrated by adding increasing amount of the protein (0–28 µM) to 10 µM of heme in 40% dimethyl sulfoxide (DMSO) buffered with 20 mM HEPES (pH 7.4). Difference in absorption spectra over a range of 350 to 700 nm was recorded. We used the increase in absorbance at Soret peak (412 nm) to monitor the formation of the protein heme complex. The heme binding curve was constructed by plotting the change in absorbance at the Soret peak (ΔA_412_) versus the protein concentrations. The heme-binding curve was fitted using *one site specific binding with Hill slope* model on GraphPad Prism, v5.00.

### Statistics

Data analysis was performed on GraphPad Prism, v5.00. Mann-Whitney test was used to compare differences between two groups, while Kruskal-Wallis test was applied to compare differences among several groups. All plotted data are means with error bars representing standard deviation (SD). Statistical significance was designated as p<0.05.

### Accession numbers

PFAM: PF01390, SCOP: 82671, SCOP: 54861, PDB: 2e7v, PDB: 2acm, PDB: 1ivz, GenBank: AY570748, GenBank: AY570737, GenBank: AY570742.

## Results

### Molecular structure model based identification of extracellular SEA-domain

We had identified a novel gene family with similar signal sequence and promoter regions among SST isolated cDNAs (Figure S1A) [26], and showed that this gene family had originated from retrotransposon-mediated gene duplication mechanism [27]. Although several transcripts from ∼27 duplicons were found to belong to this family, we could not readily identify the molecular functions of these genes since no sequence homolog was readily identifiable in any other organism [27]. Consequently, we utilized comparative structural homology modeling to identify features and domains that could predict the putative molecular functions of the encoded proteins. Firstly, protein topology indicated that while all the members of this family bear similar signal sequence and are thus trafficked to the surface; some also contain C-terminal transmembrane regions, akin to type-I transmembrane proteins (Figure S1B).

**Figure 1:**
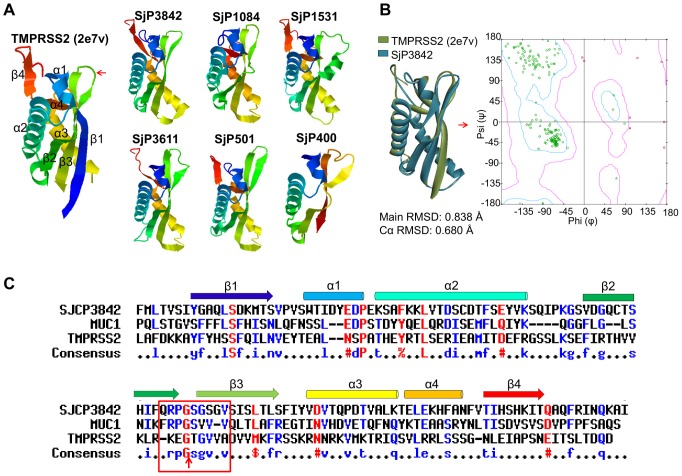
Extracellular loop of the candidate proteins contain SEA-domains. (**A**) Modeled molecular structures of the extracellular domains with striking similarity with SEA-domain. Also shown for comparison is the SEA-domain of mouse TMPRSS2. Typical of SEA-domains, the secondary structure components showed an antiparallel arrangement of β-sheets. A summary of structural models of the entire transcripts in this gene family is shown in Table S1. (**B**) Rigid body superposition of SjP3842 (blue) over the highest scoring template, PDB: 2e7v (olive). The graph is the Ramachandran plot (φ/ψ) showing conformational angles distribution of the residues. Over 98% of residues were in the favored regions while less than 2% were in the outlier region. (**C**) Alignments of SjCP3842 with two well defined SEA-domains (human MUC1 and mouse TMPRSS2). Putative SEA-domain consensus cleavage site (red arrow) was identified between β2 and β3.

The molecular folding patterns of the proteins were modeled simultaneously in *Phyre* 2 and *Rosetta* using fold recognition and *ab-initio* structure predictions (Figure S1C). These programs create sequence alignment profiles from PSI-Blasts followed by scanning of ‘fold library’ to identify remote structural homologs from experimentally determined structures in PDB and SCOP databases [33], [34]. The secondary structure components showed antiparallel arrangement of β-sheets, backed by α-helices (Figure 1A), typical of ferredoxin-like folds. Interestingly, models from both programs identified an extracellular domain of ∼120 amino acids common among this family, with striking similar folding pattern as SEA-domain (**s**ea urchin protein, **e**nterokinase and **a**grin) [PFAM: PF01390; SCOP: 82671] (Figure 1A and Table S1). SEA-domain is a domain with ferredoxin-like fold [SCOP: 54861], found in several proteins of diverse functions in different organisms [28]–[30], [41], [42]. Notably, crystal structure of the SEA-domain of transmembrane protease serine II (TMPRSS2) of *Mus musculus* [PDB: 2e7v] was the highest scoring template at over 95% confidence, according to which the shown structures were modeled. For clarity, only the original SST identified candidates are shown as representative of the family (Figure 1A). The structural models for all members of the gene family are summarized in Table S1. Other high scoring homologs at over 95% precision were the SEA-domains of Mucin 1 [PDB: 2acm] and Mucin 16 [PDB: 1ivz].

To validate the models, rigid body superposition with the highest scoring template [PDB: 2e7v] was performed. The result showed Cα and main chain root mean square deviations (RMSD) of 0.680 Å and 0.838 Å respectively for SjCP3842, a representative member of this gene family (Figure 1B). Similar low RMSD values were recorded for the other candidates. Ramachandran plot (φ/ψ) of conformation angles for each residue showed over 98% of the residues in the favored region, with less than 2% in the outlier region. These results indicate the reliability of the predicted models (Figure 1B).

A reciprocal *‘BackPhyre’* using the modeled structures to scan over 25 genomes also mapped the domain to SEA-domains at over 95% confidence, albeit with limited protein sequence homology. The low sequence similarity (Figure 1C) observed from alignments of this extracellular domain with two major SEA-domains (MUC1 and TMPRSS2) could imply that this structural similarity is at least partly independent of amino acid sequence homology [29]. As a matter of fact, SEA-domains are primarily defined by their characteristic folding pattern, extracellular localization on transmembrane proteins, their ability to assist or regulate binding to glycans, and their presence in proteins with *O*-linked glycans [28], [29], [41]. As expected, multiple *O*-glycosylation sites were identified by posttranslational modification prediction. We also confirmed that the expressed proteins contain *O*-linked glycans using glycoprotein detection assay (Figure S2). Equally, two conserved cysteine residues are present in all the candidates (Figure S1A), which could be structurally important by providing disulfide bridges in the folded protein.

Further evidence to classify the identified domain as SEA-module was the identification of the typical glycine-serine amino acid consensus (frpG/Svvv) [30] auto-cleavage site of SEA-domains (Figure 1C). Some SEA-domain proteins have been shown to undergo auto-cleavage, although the resulting subunits remain non-covalently associated in the native state [30], [41], [42]. This cleavage site is usually located within the bend between β2 and β3 sheets [30] as we equally observed (red arrow in Figure 1 A and C). In addition, the SDS fractionated recombinant protein (shown later) contained extra bands of expected molecular weight as the potential cleavage products. Taken together, these results provide multiple grounds to classify this extracellular domain as SEA-domain.

### Identification of heme-binding site on the SEA-domain

To provide lead to the possible molecular function of the gene products, we subjected the modeled structures to ligand binding site identification using *3DLigandSite* [35]. This program uses protein structure to search a structural library to identify homologous structures with bound ligands, which are then superimposed on the protein structure to predict potential ligand binding sites [35]. Interestingly, a binding site was observed for Fe-protoporphyrin-IX (heme) at significantly high precision (Figure S3). Binding sites for energy transfer coenzymes including ATP, and several metal ions (Mg, Zn, Cu) binding sites were also identified. The heme-binding site was predicted based on 178 heme ligands present in 177 homologous structures with bound heme (Figure S3).

**Figure 2:**
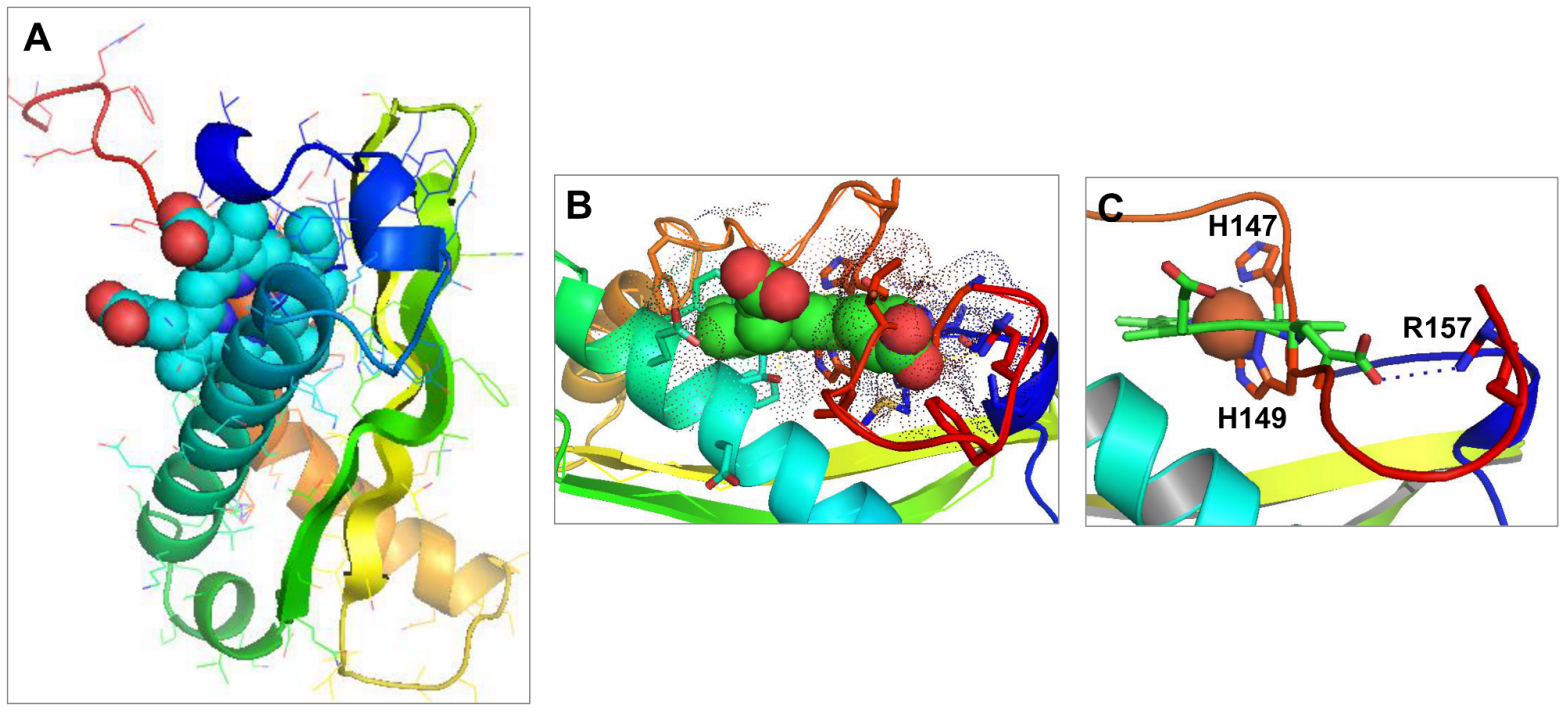
Heme-binding pocket of SjCP3842. (**A**) Heme-binding mode of SjCP3842 showing the hydrophobic vinyl end of the protoporphyrin heme inserted into a hydrophobic cavity, while hydrophilic propionate end of points away from the pocket. Heme is represented using spheres model colored by atoms (C: green, N: blue, O: red, Fe: brown). The protein is shown using cartoon model. (**B**) Heme-binding site showing the Connolly surface of the binding pocket (dots). (**C**) Heme iron (brown sphere) hexa-coordinated with His-149 and His-147 as axial ligands.

Analysis of the modeled heme-binding pocket of SjCP3842 showed that the vinyl end of the amphiphilic heme is inserted into a hydrophobic cavity created between α2 and α3 helices, and β2 and β3 sheets (Figure 2 A and B). Many of the interacting residues in the binding pocket are conserved among the members of this protein family (labeled in red in Figure S4B), consistent with binding of a heme group. The hydrophilic propionate end (red sphere) of heme is rather facing away from the hydrophobic pocket (Figure 2 A and B), with one propionate group engaged in electrostatic interactions with a nitrogen atom in Arg-157 side chain (Figure 2C). The phenyl rings of three conserved phenylalanine residues (Phe-80, Phe-140 and Phe-156) and one other phenylalanine (Phe-143) engage in pi-stacking interactions with the heme Pyrrole rings, which further stabilize heme-binding (Figure S4B). There were also polar contacts between heme and Thr-79, Tyr-83, His-147 and His-149 (Figure S4B), and several hydrogen bond interactions within the binding site.

Consistent with binding to heme, we readily identified potential axial ligands for heme iron, indicating hexa-coordination state involving two possible pairs. The imidazole group on His-149 side chain (bond distance of 2.0 Å) is the putative proximal ligand with either His-147 (Figure 2C) or the thioether group on Met-50 (Figure S4C) as the distal ligand of heme iron. However, the exact pair of axial ligands or the possibility of simultaneously binding two molecules of heme needs to be experimentally clarified. Similar binding site characteristics were observed in another characterized candidate (SjCP1531). However, the iron is coordinated to Tyr-154 as its axial ligand (Figure S5).

### Developmental stage specific expression of the candidate genes

**Figure 3:**
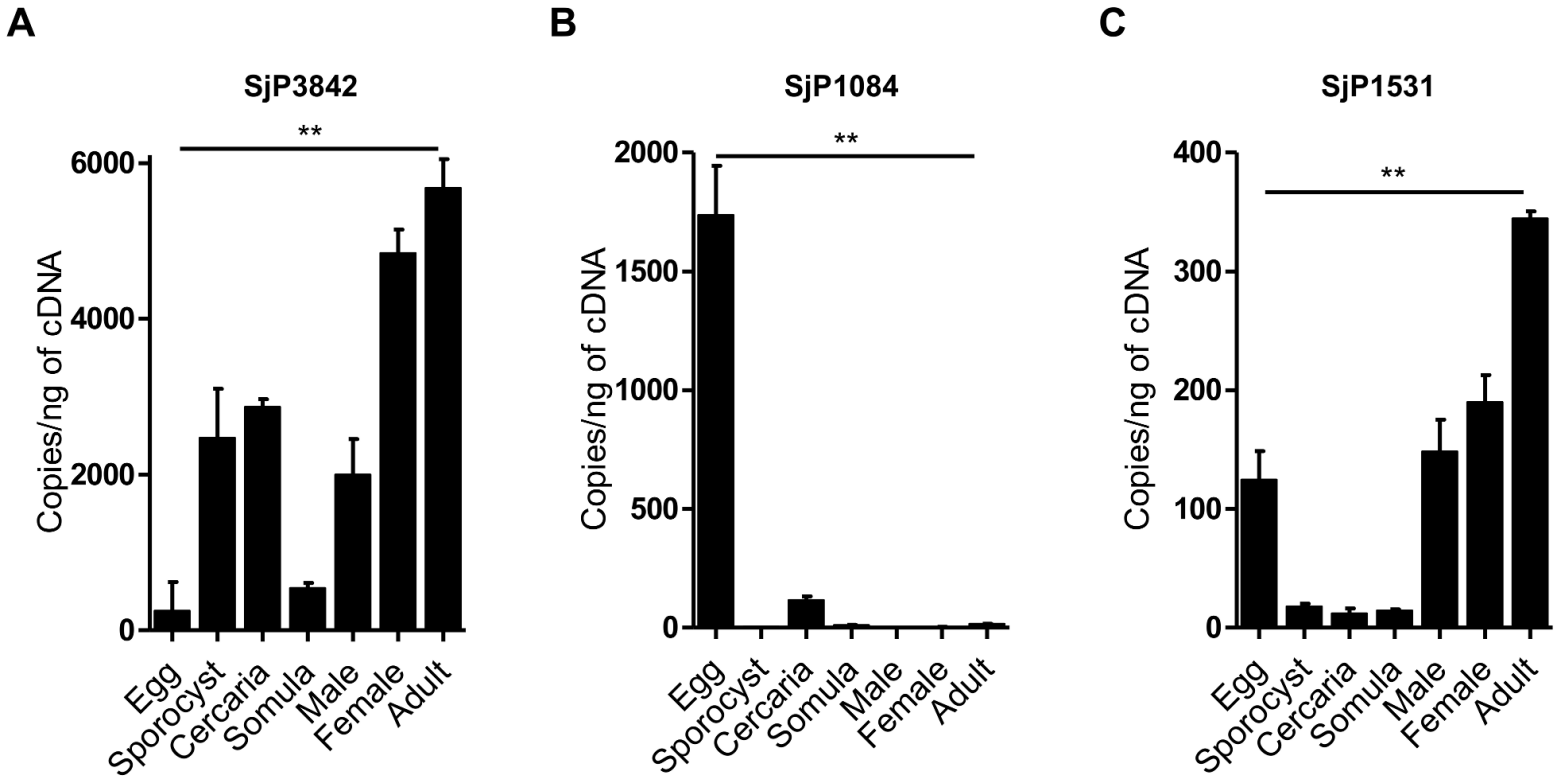
Developmental stage specific expression profiles of the candidate genes. Developmental stage specific expression of the candidate genes presented as copy number per nanogram of cDNA. The full data statistics is shown in a supplementary table (Table S2). There was differential expression of the three characterized genes among developmental stages of the parasite, with SjCP3842 expressed at higher levels relative to the other two candidates. (**A**) SjCP3842 was overtly expressed at the adult stage especially in female adult worm. The expression level at the snail intermediate sporocyst and cercaria stages were also relatively high as compared to schistosomula and egg stage. SjCP3842 was expressed at the minimal level at the egg stage. (B) Conversely, SjCP1084 was mainly expressed at the egg stage in relation to other stages. (C) The expression levels of SjCP1531 in all stages of the parasite were relatively low and mainly expressed at the egg and adult stages. Bars represent standard deviation (SD). * = p<0.05, ** = p<0.01. n = 4 for each group.

We investigated whether this gene family is differentially expressed among developmental stages of *S. japonicum* by stage specific mRNA expression using real time PCR. All other *in-vitro* based characterization was limited to three candidates: SjCP3842 [GenBank: AY570748], SjCP1084 [GenBank: AY570737] and SjCP1531 [GenBank: AY570742]. Relative expression of each candidate gene was quantified and expressed as copy number per nanogram of cDNA (Figure 3 and Table S2). There was differential expression of the three genes among developmental stages of the parasite, with SjCP3842 expressed at higher levels relative to the other two characterized candidates (Figure 3 and Table S2). SjCP3842 was overtly expressed in the adult stage (5680±370.9), although at a higher level in female adult worm (4846±302.1) as compared to the male worms (2000±453.9). The expression levels in the snail intermediate inhabiting sporocyst (2474±627.2) and infective cercaria (2871±98.4) stages were also relatively high as compared to somula (543.4±64.1) and egg stage (252±370.1). SjCP3842 was expressed at the minimal level in the egg stage (Figure 3A). Conversely, SjCP1084 was mainly expressed in the egg stage in relation to other stages. However, the expression levels of SjCP1531 in all stages of the parasite were relatively low and mainly expressed at the egg and adult stages (Figure 3C and Table S2).

### Cloning, recombinant expression and antigenicity of the candidates

**Figure 4:**
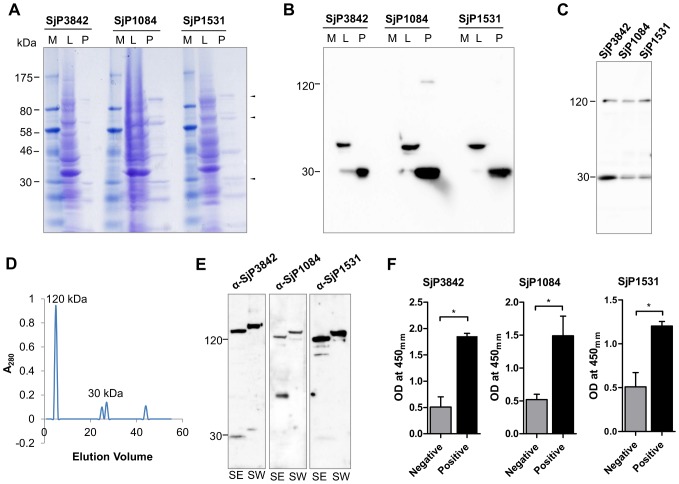
Protein expression and antigenicity of the candidate proteins. (**A**) SDS-PAGE of recombinant *E. coli* lysates (lane L) and purified protein without inclusion bodies (lane P). Arrows indicate expected molecular weights of oligomers. Other bands are expected SEA-domain cleavage products. (**B**) Western blots of recombinant protein expression as in (B), probed with anti-HisG antibody. (**C**) Anti-HisG probed western blots showing oligomerization of proteins with multiple bands of additive ∼30 kDa subunits, and tetramer as the most stable state. (**D**) Size exclusion gel filtration chromatography of SjCP3842 showed multiple elution peaks, another evidence of oligomerization. (**E**) Immunoblots showing reactivity of parasite crude antigen preparations (SEA and SWA) with immune sera. (**F**) The candidate proteins specifically reacted with infected miniature pig sera in IgG ELISA, indicating potential antigenicity during schistosomiasis. Bars represent standard deviation (SD). * = p<0.05, ** = p<0.01. n = 4 for each group.

To confirm expression at protein level, we expressed recombinant proteins, generated and used specific immune sera to identify the native proteins in parasite crude extracts. The complete coding regions of the genes were amplified from *S. japonicum* adult worm cDNA library and cloned into the expression vector, pcDNA4-HisMax. For recombinant protein expression, the plasmid constructs were transformed into Freestyle 293 and BL21 *E. coli* cells. The recombinant proteins used for biochemical assays were expressed in Freestyle 293 cells to ensure proper folding and post translational modification. The proteins were found to exist as oligomers in the native state as seen in the multiple bands of additive ∼30 kDa subunits observed both on SDS-PAGE (Figure 4A), western blots probed with anti-HisG antibody (Figure 4 B and C), and by multiple peaks from size exclusion chromatography fractions (Figure 4D), all showing the tetramer as the native state. Similar oligomeric state was also predicted by structural modeling (Figure S1D). Oligomerization may have been mediated by the disulfide bridges on two conserved cysteine residues common among the members of this family (Figure S1A). Other extra bands are of same molecular weight as the expected SEA-domain auto-cleavage products (Figure 1C).

To confirm native expression and to show potential antigenicity of the candidates during schistosomiasis, immunoblotting and ELISA techniques were applied. Parasite egg (SEA) and adult worm (SWA) crude antigen preparations were blotted and probed with the polyclonal immune sera (α-SjCP3842, α-SjCP1531 and α-SjCP1084). Blotted protein fractions of sizes similar to both the subunits (∼30 kDa) and tetramer (∼120 kDa) reacted specifically with the immune sera (Figure 4E). Also, the recombinant proteins specifically reacted with sera from *S. japonicum* infected miniature pigs, with significantly high titers of IgG in ELISA (Figure 4F). These results indicate that this gene family is actually expressed in the parasite, appear functional and potentially antigenic during schistosomiasis.

### SEA-domains of *S. japonicum* assist binding to glycosaminoglycans (GAGs)

**Figure 5:**
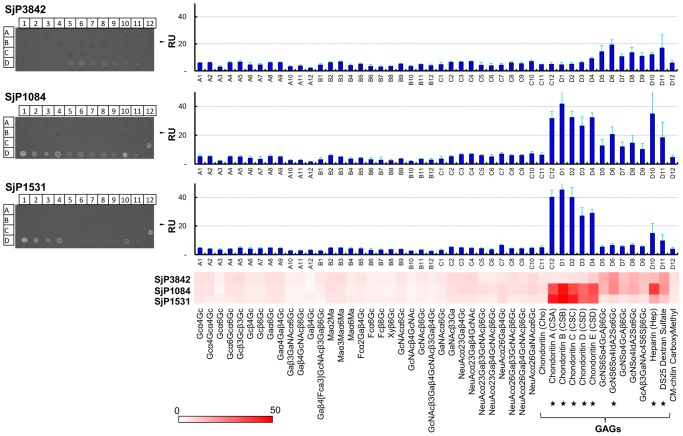
*S. japonicum* SEA-domains mediate binding to glycosaminoglycans (GAGs). Interactions between glycans and SEA-domain proteins were analyzed using array type sugar chip in SPR system. Shown here are the SPR imaging and SPR signals (RU), which is proportional to the amount of proteins bound to glycans immobilized on sensor chips in an array format. There was high-affinity binding to chondroitin sulfate, heparin, dextran sulfate and other sulfated GAGs. The binding kinetics is shown in Figure S6.

In addition to their characteristic folding pattern, SEA-domains are known to assist or regulate binding to carbohydrate moieties. We assessed interactions of the characterized SEA-domain proteins with glycans using recombinant proteins and array type sugar chips in a Surface Plasmon Resonance (SPR) system [37]. The SPR signal (expressed in resonance units, RU) is proportional to the amount of protein analytes bound to the sugar chains immobilized on the sensor chip in a 48 glycans array. The SPR imaging showed specific binding to sulfated GAGs with relatively high affinity. There was disproportionately high specific binding to chondroitin sulfate, dermatan sulfate (CS-B), heparin, dextran sulfate and other sulfated GAGs (Figure 5). SjCP1084 and SjCP1531 have similar glycan binding pattern while SjCP3842 showed relatively less glycan binding capacity but also preferentially binds sulfated GAGs (Figure 5).

We further confirmed the specificity and affinity of protein-GAG interactions by using chondroitin sulfate GAG (CS-GAG) chip containing all possible sulfated disaccharides subunits of chondroitin sulfate, and different concentrations of the protein as analytes. The glycan array format of the CS-GAG chip used and the SPR imaging of the glycan binding assays are shown in a supplementary file (Figure S6 A and B). The binding kinetics of the carbohydrate-protein interactions showed significant binding affinity to CS-GAGs, with dissociation constant (*K*_D_) within the range of receptor-ligand interactions (Figure S6 C and D). Figure S6C shows the detailed sensorgram and the binding curve of the interaction between SjCP1084 and chondroitin sulfate E (*K*_D_ = 9.84×10^−9^ M), as representative of the binding kinetics data. The other *K*_D_ values for the interactions of SjCP1084 and SjCP1531 with different sulfated disaccharides of chondroitin sulfate are summarized in Figure S6D, showing values within nanomolar range. These results indicate the specificity and affinity of the observed protein-glycan interactions.

### Heme-binding properties of *S. japonicum* SEA-domain proteins

**Figure 6:**
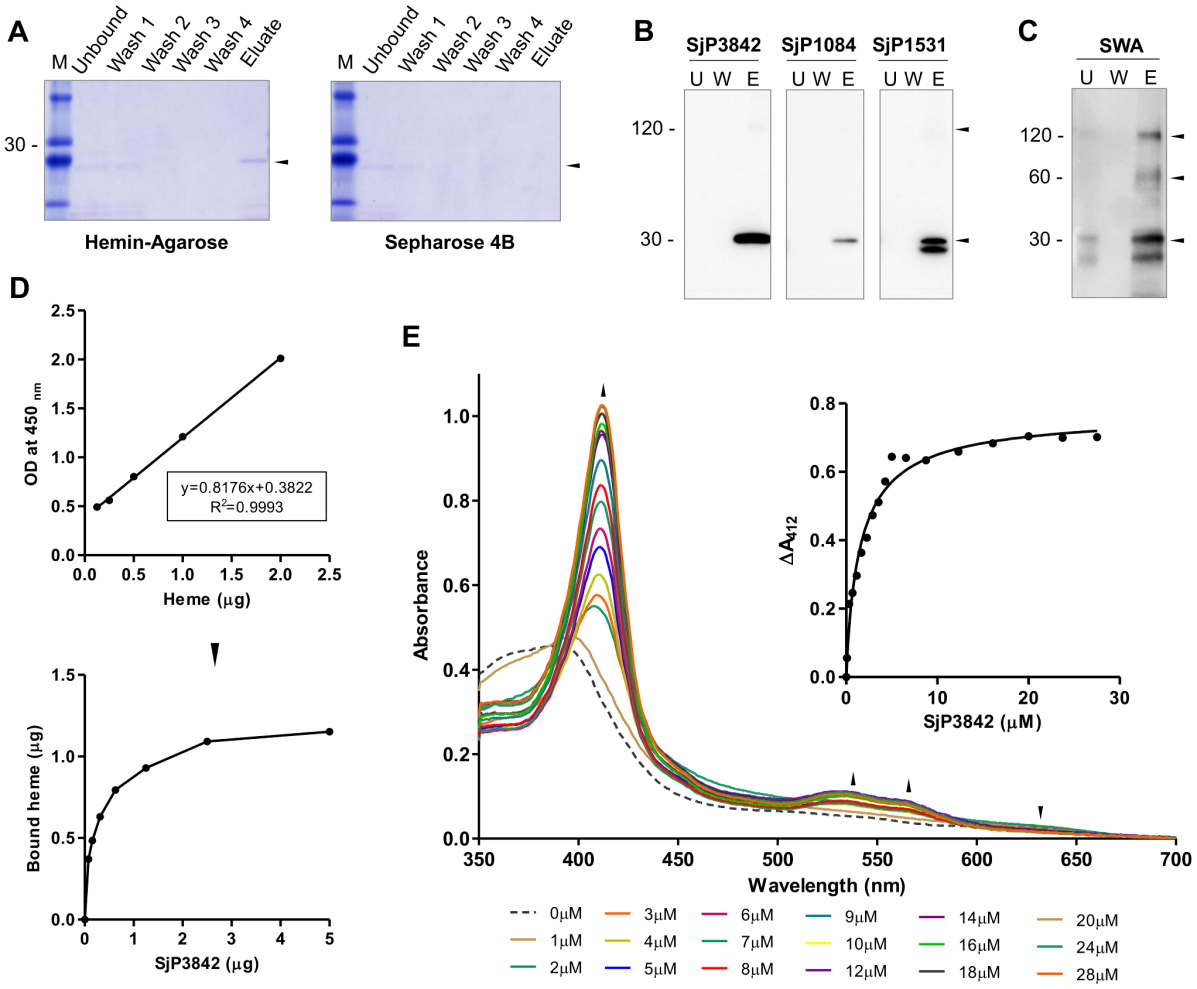
*S. japonicum* SEA-domain proteins are heme-binding proteins. (**A**) Hemin-agarose binding assay showing potential of SjCP3842 to bind heme on hemin-agarose beads. (**B**) Hemin-agarose binding assay confirmed by immunoblotting using three candidates. ‘U’: unbound, ‘W’: last wash, ‘E’: eluates. (**C**) Identification of SjCP3842 in heme-binding protein fractions from parasite crude extracts (SWA). (**D**) Estimation of the amount of heme bound using peroxidase activity of bound heme. Standard curve (linear graph) of peroxidase activity of known concentrations of hemin was used to estimate the amount of bound heme. (**E**) Differential spectral titration of protein-heme interaction using 10 µM of heme and increasing concentrations of the protein (0 to 28 µM). Soret peak was red shifted from 388 nm to 412 nm, and absorption maximum increased with increasing accumulation of protein-heme complex. The inset is the heme-binding curve constructed by plotting ΔA_412_ versus protein concentration, showing 1∶1 stoichiometry.

To validate the structure based heme-binding model, we showed heme-binding properties of this family *in-vitro*, by three independent methods: hemin-agarose binding assay, heme-dependent peroxidase activity of protein-hemin complexes and optical UV absorption spectroscopy. First, we showed using SjCP3842 that the purified recombinant protein has potential to bind heme on hemin-agarose beads. The eluted fraction showed evidence of specific binding of the protein to heme (Figure 6A). Same experiment performed using unconjugated Sepharose 4B as negative control did not show any trace of the protein in the eluted fraction. Heme binding assay was repeated using the three characterized candidates and similar specific binding was consistently observed after immunoblotting using immune sera (Figure 6B).

To confirm this observation in the native state, hemin-agarose beads were incubated with parasite adult worm crude antigen (SWA) to isolate the total heme-binding protein fractions in the parasite. The fractions were blotted and probed with monoclonal antibody against SjCP3842 (Figure 6C). The result clearly showed the presence of the protein in the parasite heme-binding protein fractions. The multiple bands are expected molecular weights of the monomer, dimer and tetramer. The fact that binding was ablated by the reducing effect of β-mercaptoethanol and denaturing effect of sodium dodecyl sulfate (SDS) used for elution suggests that the observed heme-binding property is at least partly non-covalently mediated by structure of the folded proteins.

To estimate the amount of heme bound by the protein, we assayed the heme-dependent peroxidase activity of the protein-hemin complex using SjCP3842. We first estimated the peroxidase activities of known concentrations of hemin, and used the resulting standard curve (linear graph) to estimate the amount of heme bound by the characterized heme-binding protein based on the peroxidase activity of bound heme (Figure 6D). The result showed that the amount of bound heme increased with increasing protein concentration, reaching saturation at about 2 µg of protein, when 1 µg of hemin was bound (Figure 6D).

To further assess the binding affinity of the protein-heme interaction, optical absorption spectra of the protein-heme complex was monitored by differential titration of 10 µM of heme with increasing concentrations of the protein (0 to 28 µM) (Figure 6E). The Soret absorption peak for heme alone was characteristically broad and was initially 388 nm prior to addition of the protein (broken lines). The Soret absorption maximum was red shifted to 412 nm on addition of protein and absorbance at this peak increased gradually depending on accumulation of protein-heme complex, until saturation at about 1∶1 molar ratio. The Q-bands (534 nm and 564 nm) and the isobestic points were also apparent, indicating the presence of two absorbing species (heme and protein-heme complex) in the solution. The UV-visualization spectral attributes of the protein-heme complex (Soret peak, 412 nm; Q-bands, 534 nm and 564 nm) were typical of heme with hexa-coordinated ferric iron [40], [43], consistent with the structural model of this study. However, this needs to be confirmed by electron spin resonance spectroscopy. The inset is the heme-binding curve constructed by plotting ΔA_412_ versus protein concentration (Figure 6E). The curve fitting indicates increasing accumulation of the protein-heme complex with saturation after about 10 µM of protein was added, thus suggesting a 1∶1 stoichiometry. The fitting yielded equilibrium dissociation constant *K*_D_ = 1.605×10^−6^ M, indicating high affinity for binding heme. Taken together, these observations confirm the potential of the novel SEA-domain proteins to specifically interact with heme.

### Localization on adult worm teguments and gastrodermis

**Figure 7:**
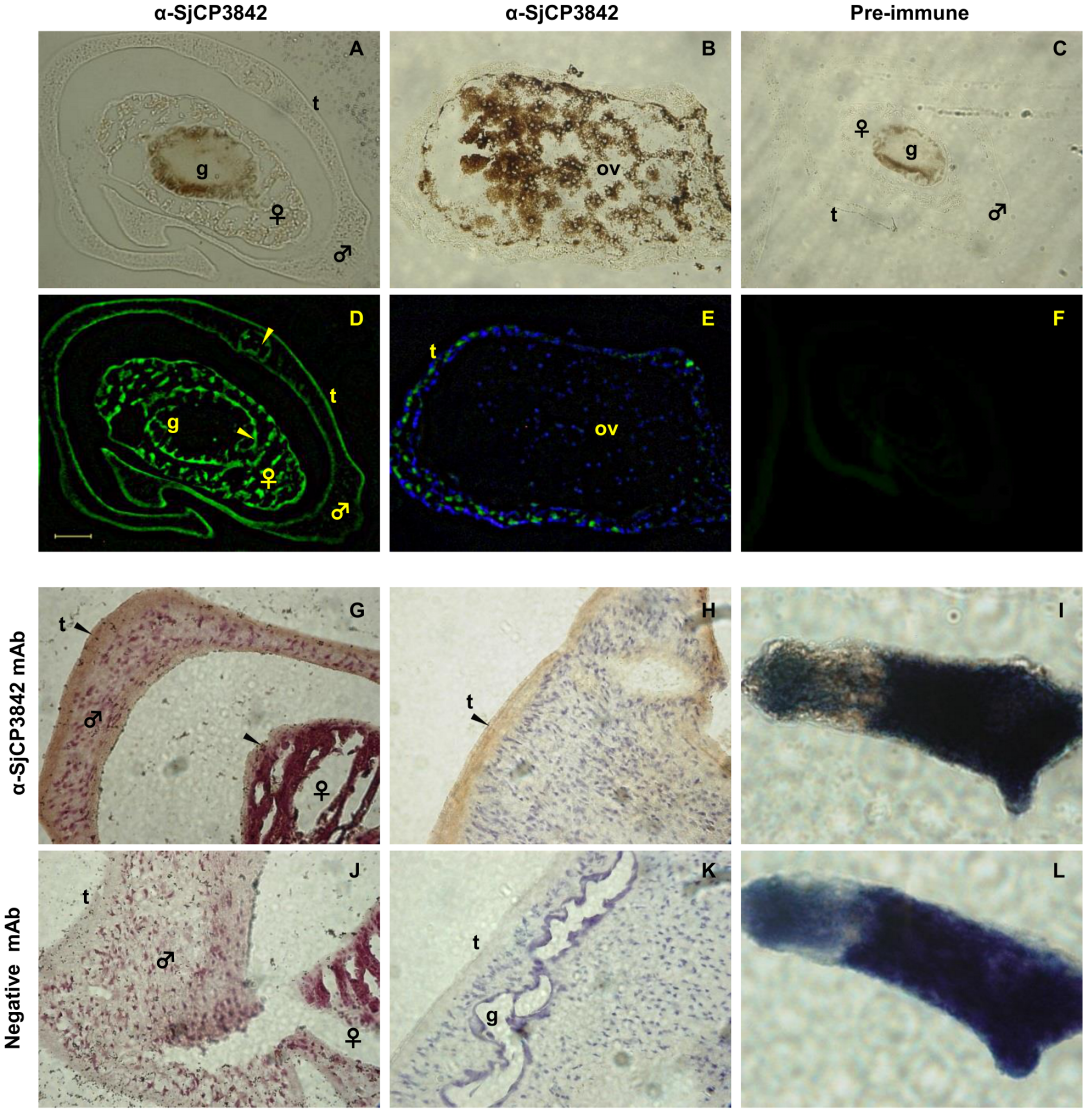
Immunolocalization on the teguments and gastrodermis. Tissue localization of native SjCP3842 was shown using immunofluorescence (A–F) and immunoperoxidase (G–L) methods. (**A**) Bright field image of cross section of adult worm pair. (**B**) Bright field image of cross section of female adult worm showing the ovary. (C) Bright field image of a cross section of adult worm pair for control IFA. (**D**) IFA on a cross section of adult worm pair using monoclonal antibody showed that the native SjCP3842 was localized on the adult worm tegument (t) and gastrodermis (yellow arrows) of the parasites gut (g). Scale bar = 50 µm. (**E**) IFA on a cross section of a female worm showing FITC staining of SjCP3842 on adult worm teguments but not in the content of the ovary. DAPI staining of nuclei was detected in the tissue and ovary. (**F**) IFA using pre-immune serum as negative control. (**G** and **H**) Immunoperoxidase detection (brown) of SjCP3842 in a section of adult worm pair showed localization on adult worm tegument. (**I**) Immunoperoxidase localization of SjCP3842 on juvenile schistosomula showed localization on the tegument. Negative immunoperoxidase detection was observed in sections of adult worm (**J** and **K**) and schistosomulae (**L**) probed with pre-immune serum.

To ascertain the tissue distribution of the products of this gene family in the parasite, immunolocalization was performed by immunofluorescence assay (IFA) and immunoperoxidase staining. For clarity and because similar tissue localization patterns were observed, only the data for SjCP3842 is shown here. The results for the other candidates are presented in a supplementary figure (Figure S7). IFA on adult worm sections showed that the native SjCP3842 was localized on the adult worm tegument and gastrodermis of the parasites gut (Figure 7 A and D). Similar results were observed for all the three candidates as presented in a supplementary figure (Figure S7). No signal was observed in the ovary as shown in the cross section of the female adult worm probed with anti-SjCP3842 monoclonal antibody (Figure 7 B and E), which is consistent with minimal expression in the egg as earlier shown in the developmental stage specific gene expression (Figure 3A). The nuclei are stained with DAPI, showing staining both in the parasite tissues and the content of the ovary. No signal was observed in the sections incubated with sera obtained from control mice (Figure 7 C and F).

Equally, immunolocalization was repeated using immunoperoxidase-DAB technique with biotinylated monoclonal antibody detected with streptavidin-HRP. The result again showed localization on the adult worm teguments (Figure 7 G and H). The protein was also found localized on the tegument of the juvenile schistosomula stage (Figure 7I). No peroxidase activity was detected in the sections probed with pre-immune serum (Figure 7 J–L). Taken together, these results indicate localization on adult worm teguments and gastrodermis, and schistosomula teguments.

## Discussion

We have utilized comparative homology modeling to identify remote structural homologs, and successfully characterized a novel gene family encoding SEA-domain proteins from *S. japonicum*. Similar strategy was used to identify and characterize heme-binding property for this domain, thereby providing insight into the potential biological function of otherwise ‘hypothetical proteins’. Functional annotation of proteins routinely relies on sequence homology with already characterized proteins or at least domains with experimentally resolved functions. However, the degree of evolutionary conservation of the structural architecture of proteins is greater than the amino acid sequence conservation [24], [25]. Our results affirmed that absolute reliance on sequence homology for functional annotation of proteins is not exhaustive. In the post-genome era, the vast accumulation of sequence data has opened new frontiers for identification of intervention targets. However, determination of protein functions is one of the major challenges since sequence homology alone has proven insufficient for placing the vast amount of ‘*omics*’ data into functional context [24], [25]. It is necessary to explore other strategies that can effectively identify remote homologs, which are not readily identifiable from sequence data. The data presented here is a typical example of the possible application of molecular structural analysis to identify and characterize novel protein functions.

Like most previously characterized SEA-domain containing proteins, our candidates specifically interacted with sugar chains, especially glycosaminoglycans (GAGs) [28], [41]. GAGs are long linear polysaccharides composed of repeating disaccharide units, usually linked covalently to a core protein to form a proteoglycan. While the protein core keeps the proteoglycan localized on the cell surface or in the extracellular matrix (ECM), the GAGs components mediate interactions with a plethora of extracellular ligands and effectors. All cellular processes that involve cell surface molecular interactions including: ligand-receptor, cell-cell and cell-matrix interactions, will likely involve proteoglycans and GAGs because these molecules are ubiquitous and are shown to functionally bind proteins to regulate important developmental processes [44]–[46].

In addition to their space filling and organizational roles in the ECM, GAGs on proteoglycans can modulate the function of a repertoire of extracellular effectors by their roles in: ligand gathering, clustering and oligomerization of ligands and their receptors [45], [46], and their ability to act as storage depots for ligands by sequestering them and preventing their rapid degradation [45]. Proteoglycans are required as co-receptors for some growth factors and cytokines signaling in collaboration with the cognate signaling receptors in a ligand-receptor-proteoglycan ternary complex [45]–[47], and can also signal independently as a receptor via its cytoplasmic domain [47], [48]. Proteoglycans can also undergo proteolytic cleavage near the plasma membrane to shed their ectodomain as soluble regulators [49]. Specific interaction with GAGs of host (*trans*) or parasite (*cis*) origin as we observed here may suggest some functional role of this protein family as parasite receptors for accessing ligands and signals, especially of host origin. From the foregoing, and given that *S. japonicum* genome encodes many receptors and signaling molecules but sometimes not the ligands [21], it is plausible that parasite membrane receptors with GAG-binding potential could interact with its own or host proteoglycans in a receptor-proteoglycan-ligand ternary complex [45]–[47], as a means of accessing host molecules tethered on GAGs for signals for their growth, development, and maturation thus rendering them potential intervention targets.

The native proteins were localized at the parasite tegument and gastrodermis, sites that are of immunological significance being located at the host-parasite interface [14], [16]. These sites are rich in proteins that are often unique to schistosomes, some of which can directly interact with host derived molecules as observed in the characterized SEA-domain proteins [14], [16]. The ability of the parasite to bind GAGs on host secreted or shed proteoglycans [49] or proteoglycans on the surface of host immune cells [50] could result in masking of the ‘non-self’ status of the parasite, thereby evading attack by host immune system [51]. It is thus possible from the foregoing, that this gene family could also be involved in some immune evasion mechanisms. We are presently targeting the candidates that are expressed at the infective cercarial, schistosomula and adult stages for possible vaccine application.

Heme-binding properties have been described here for the first time for SEA-domain proteins from this hemophagous parasite. In terms of the parasite biology and host-parasite interaction, this finding represents a significant contribution towards elucidating heme detoxification and heme iron acquisition mechanisms of the parasite. Schistosomes inhabit the hepatoportal veins of the host, where they feed on host erythrocytes and catabolize the globin moieties of hemoglobin as a major source of the requisite amino acids for their growth, development and reproduction [3], [4]. However, the released heme moiety is potentially toxic due to its reactive nature and ability to produce free radical species, lipid peroxidation, and protein and DNA oxidation [3], [6]. Hemophagy-adapted parasites have therefore evolved strategies to sequester and detoxify heme [3]–[9]. Heme iron is arguably the major source of iron for this parasite, thus, the parasite also maintains a heme acquisition mechanism to harness the needed iron from heme molecules [4], [10]. These candidates are localized on adult worm gastrodermis, the site for heme detoxification [8], [17] and acquisition [3], [10]; and in the adult and schistosomula teguments, also potential sites for heme acquisition in these stages [20]. Indeed, effective heme homeostasis mechanism is paramount for parasite survival and establishment, and is a major target of effective drugs against hemoparasites including the *quinines* and *artemisinine* [11]–[13]. Unfortunately, the exact mechanisms and the molecules involved in heme-homeostasis are still controversial. However, there is a consensus on the involvement of heme-binding proteins both as nucleation agents for heme crystallization [6]–[8], [17], and as surface heme receptors in an ABC- (ATP binding cassette) transporters coupled heme uptake mechanism [38], [52], [53].

The developmental stage specific expression, especially of SjCP3842, clearly showed overt expression at the adult stage especially the female adult worms, which is consistent with the heme homeostasis requirements of this stage. There was also relatively high expression in the snail inhabiting sporocysts and the infective cercariae. The observation that the sporocysts also express this gene indicates expression at the snail stage as well, which may suggest similar or different function in the snail host. With regards to heme binding function, the sporocysts are known to absorb nutrients from snail host through their tegument for nourishment of cercariae in their germinal sac [54], and heme binding proteins have also been identified among secreted proteins from the sporocyst stage [55]. Since iron source in snail is mainly in the form of heme, it is plausible that heme binding proteins like the ones we characterized might be required for heme iron uptake from snail hosts, as well as other functions. SEA domain still do not have a well characterized function apart from interaction with glycans (GAGs), to which we and others have alluded several potential implications like ligand acquisition and immune evasion. The prospect that this gene family could perform more than one function in different developmental stages of the parasite implies that hemophagy might have been a major factor among other selection factors for this gene family.

SEA-domains are characteristically found in carbohydrate rich mucous environments [30]. The heme-binding SEA-domain proteins we described here are localized in the parasite gastrodermis and tegument. The gastrodermis is the syncytial linings of the parasite gut, the site for hemoglobin catabolism, heme sequestration, detoxification and acquisition. A similar structure called peritrophic matrix (PM) with heme-binding property has been described in the midgut of hemophagous insects. The PMs perform a central role in heme homeostasis by protecting the insects' midgut against damage from heme toxicity [56], akin to schistosomes gastrodermis. The PMs are complex matrices composed of heme-binding proteins, proteoglycans, chitins and chitin-binding proteins [56]. Specifically, *Aedes aegypti* Mucin I (*Ae*MUC1) was identified as a major heme-binding protein in the PM [57]. MUC1 and the proteins we characterized here both contain SEA-domains. It is therefore plausible that similar mechanism mediated by heme-binding SEA-domain proteins may exist in schistosomes' gastrodermis. However, this hypothesis will need to be experimentally clarified by isolating and identifying all heme-binding proteins of the parasite and/or the parasite gastrodermis. We will design further studies to fully characterize the role of this gene family in the parasite heme-homeostasis and heme acquisition mechanisms, and explore prospects for its application in disease intervention.

## Supporting Information

Figure S1 **Multiple alignments and molecular structures of the SEA-domain proteins from** ***S. japonicum*** **.** (**A**) Multiple alignments of SST isolated candidates showing similar signal sequence [27]. (**B**) The Topology of the members of the gene family. (**C**) The modeled molecular structures of whole molecules showing the SEA-domain. (**D**) Predicted oligomeric state of SjCP3842 as a representative of the family.
(TIF)

Figure S2 **Glycoprotein detection analysis showed evidence of** ***O*** **-linked glycosylation in the expressed candidate proteins.** We showed using glycosylation detection assay that the expressed proteins contain *O*-linked glycans. Glycosylated proteins were detected as magenta stained bands in SDS-PAGE fractions.
(TIF)

Figure S3 **Molecular structure based identification of heme-binding site.** 3DLigandSite ligand binding site prediction report showing structure based identification of heme-binding site at highly significant precision. The heme-binding site was predicted based on 178 ligands present in 177 homologous structures.
(PDF)

Figure S4 **Heme-binding pocket of SjCP3842.** (**A**) Heme-binding mode of SjCP3842 showing the hydrophobic vinyl end of heme inserted into a hydrophobic cavity, while hydrophilic propionate end points away from the pocket. Heme is represented using spheres model colored by atoms (C: green, N: blue, O: red, Fe: brown). (**B**) The interacting residues in the heme-binding site. Strictly conserved residues are labeled in red while partly conserved residues are labeled in blue. Heme molecule is shown with nb-spheres model while residues are shown with sticks model, both colored by atoms. (**C**) The second predicted heme-binding mode showing heme iron hexa-coordinated with His-149 and Met-50 as axial ligands.
(TIF)

Figure S5 **Heme binding pocket of SjCP1531.** Similar binding site characteristics as observed for SjCP3842 were observed for SjCP1531. However, heme iron is coordinated to Tyr-154 as its axial ligand. (**A**) Heme-binding mode of SjCP1531 showing the hydrophobic vinyl end of the protoporphyrin heme inserted into a hydrophobic cavity, while hydrophilic propionate ends of points away from the pocket. Heme is represented using spheres model colored by atoms (C: green, N: blue, O: red, Fe: brown). The protein is shown using cartoon model. (**B**) Heme-binding site showing the Connolly surface of the binding pocket (dots). (**C**) Heme iron (brown sphere) putatively coordinated to Tyr-154 as axial ligand.
(TIF)

Figure S6 **Specificity and affinity of protein-glycan interactions.** (**A**) Array format of CS-GAG chip. (**B**) SPR imaging of the glycan-binding assay are shown. (**C**) Sensorgram and binding curve of the interaction between SjCP1084 and chondroitin sulfate-E as representative of the binding kinetics data. (**D**) Summary of equilibrium dissociation constants (*K*_D_) of protein-glycan interactions, showing values within nanomolar range.
(TIF)

Figure S7 **Immunolocalization on the teguments and gut epithelial linings.** Immunolocalization of SjCP1084 (**A** and **D**) and SjCP1531 (**B** and **E**) using IFA on cross sections of adult worm pairs probed with immune sera and detected with FITC conjugated secondary antibody. Immunoperoxidase detection of SjCP1084 (**G**) and SjCP1531 (**H**) on the juvenile schistosomulae using immune sera and HRP conjugated secondary antibodies also showed localization on the tegument. No signal was detected in adult worm sections and schistosomulae probed with the pre-immune serum (**C, F** and **I**).
(TIF)

Table S1 **Summary of structural homology modeling results for** ***S. japonicum*** **SEA-domain gene family.** In addition to the structural homology modeling data presented in Figure 1, the structural modeling was equally performed for all identified transcripts in this gene family and the result is summarized in this table.
(DOCX)

Table S2 **Developmental stage specific expression of** ***Schistosoma japonicum*** **SEA-domain containing genes.** The developmental stage specific expression of the candidate genes expressed as copy number per nanogram of cDNA. The detailed data statistics of the data plotted in Figure 3 is reproduced here to show mean values and standard deviations of each candidate at each developmental stage of the parasite.
(DOCX)

## References

[1] Chitsulo L, Engels D, Montresor A, Savioli L (2000) The global status of schistosomiasis and its control. Acta Trop 77: 41–51. 10996119 10.1016/s0001-706x(00)00122-4 PMC5633072

[2] Tran MH, Pearson MS, Bethony JM, Smyth DJ, Jones MK, et al (2006) Tetraspanins on the surface of Schistosoma mansoni are protective antigens against schistosomiasis. Nat Med 12: 835–840. 16783371 10.1038/nm1430

[3] Toh SQ, Glanfield A, Gobert GN, Jones MK (2010) Heme and blood-feeding parasites: friends or foes? Parasit Vectors 3: 108. 21087517 10.1186/1756-3305-3-108 PMC2999593

[4] Glanfield A, McManus DP, Anderson GJ, Jones MK (2007) Pumping iron: a potential target for novel therapeutics against schistosomes. Trends Parasitol 23: 583–588. 17962074 10.1016/j.pt.2007.08.018 PMC2756500

[5] Paiva-Silva GO, Cruz-Oliveira C, Nakayasu ES, Maya-Monteiro CM, Dunkov BC, et al (2006) A heme-degradation pathway in a blood-sucking insect. Proc Natl Acad Sci U S A 103: 8030–8035. 16698925 10.1073/pnas.0602224103 PMC1472424

[6] Graça-Souza AV, Maya-Monteiro C, Paiva-Silva GO, Braz GR, Paes MC, et al (2006) Adaptations against heme toxicity in blood-feeding arthropods. Insect Biochem Mol Biol 36: 322–335. 16551546 10.1016/j.ibmb.2006.01.009

[7] Chugh M, Sundararaman V, Kumar S, Reddy VS, Siddiqui WA, et al (2013) Protein complex directs hemoglobin-to-hemozoin formation in Plasmodium falciparum. Proc Natl Acad Sci U S A 110: 5392–5397. 23471987 10.1073/pnas.1218412110 PMC3619337

[8] Oliveira MF, d'Avila JC, Torres CR, Oliveira PL, Tempone AJ, et al (2000) Haemozoin in Schistosoma mansoni. Mol Biochem Parasitol 111: 217–221. 11087932 10.1016/s0166-6851(00)00299-1

[9] Egan TJ (2008) Haemozoin formation. Mol Biochem Parasitol 157: 127–136. 18083247 10.1016/j.molbiopara.2007.11.005

[10] Rao AU, Carta LK, Lesuisse E, Hamza I (2005) Lack of heme synthesis in a free-living eukaryote. Proc Natl Acad Sci U S A 102: 4270–4275. 15767563 10.1073/pnas.0500877102 PMC555530

[11] Corrêa Soares JB, Menezes D, Vannier-Santos MA, Ferreira-Pereira A, Almeida GT, et al (2009) Interference with hemozoin formation represents an important mechanism of schistosomicidal action of antimalarial quinoline methanols. PLoS Negl Trop Dis 3: e477. 19597543 10.1371/journal.pntd.0000477 PMC2703804

[12] Oliveira MF, d'Avila JC, Tempone AJ, Soares JB, Rumjanek FD, et al (2004) Inhibition of heme aggregation by chloroquine reduces Schistosoma mansoni infection. J Infect Dis 190: 843–852. 15272414 10.1086/422759

[13] Kumar S, Guha M, Choubey V, Maity P, Bandyopadhyay U (2007) Antimalarial drugs inhibiting hemozoin (beta-hematin) formation: a mechanistic update. Life Sci 80: 813–828. 17157328 10.1016/j.lfs.2006.11.008

[14] Van Hellemond JJ, Retra K, Brouwers JF, van Balkom BW, Yazdanbakhsh M, et al (2006) Functions of the tegument of schistosomes: clues from the proteome and lipidome. Int J Parasitol 36: 691–699. 16545817 10.1016/j.ijpara.2006.01.007

[15] Delcroix M, Medzihradsky K, Caffrey CR, Fetter RD, McKerrow JH (2007) Proteomic analysis of adult S. mansoni gut contents. Mol Biochem Parasitol 154: 95–97. 17451823 10.1016/j.molbiopara.2007.03.008 PMC2732360

[16] Mulvenna J, Moertel L, Jones MK, Nawaratna S, Lovas EM, et al (2010) Exposed proteins of the Schistosoma japonicum tegument. Int J Parasitol 40: 543–554. 19853607 10.1016/j.ijpara.2009.10.002

[17] Corrêa Soares JB, Maya-Monteiro CM, Bittencourt-Cunha PR, Atella GC, Lara FA, et al (2007) Extracellular lipid droplets promote hemozoin crystallization in the gut of the blood fluke Schistosoma mansoni. FEBS Lett 581: 1742–1750. 17418143 10.1016/j.febslet.2007.03.054

[18] Chen MM, Shi L, Sullivan DJ (2001) Haemoproteus and Schistosoma synthesize heme polymers similar to Plasmodium hemozoin and beta-hematin. Mol Biochem Parasitol 113: 1–8. 11254949 10.1016/s0166-6851(00)00365-0

[19] Stiebler R, Soares JB, Timm BL, Silva JR, Mury FB, et al (2011) On the mechanisms involved in biological heme crystallization. J Bioenerg Biomembr 43: 93–99. 21301942 10.1007/s10863-011-9335-x

[20] Clemens LE, Basch PF (1989) Schistosoma mansoni: effect of transferrin and growth factors on development of schistosomula in vitro. J Parasitol 75: 417–421. 2786065

[21] Consortium SjGSaFA (2009) The Schistosoma japonicum genome reveals features of host-parasite interplay. Nature 460: 345–351. 19606140 10.1038/nature08140 PMC3747554

[22] Hu W, Yan Q, Shen DK, Liu F, Zhu ZD, et al (2003) Evolutionary and biomedical implications of a Schistosoma japonicum complementary DNA resource. Nat Genet 35: 139–147. 12973349 10.1038/ng1236

[23] Liu F, Cui SJ, Hu W, Feng Z, Wang ZQ, et al (2009) Excretory/secretory proteome of the adult developmental stage of human blood fluke, Schistosoma japonicum. Mol Cell Proteomics 8: 1236–1251. 19299421 10.1074/mcp.M800538-MCP200 PMC2690496

[24] Ebihara A, Okamoto A, Kousumi Y, Yamamoto H, Masui R, et al (2005) Structure-based functional identification of a novel heme-binding protein from Thermus thermophilus HB8. J Struct Funct Genomics 6: 21–32. 15965735 10.1007/s10969-005-1103-x

[25] Sivashankari S, Shanmughavel P (2006) Functional annotation of hypothetical proteins - A review. Bioinformation 1: 335–338. 17597916 10.6026/97320630001335 PMC1891709

[26] Yu C, Zhang F, Yin X, Kikuchi M, Hirayama K (2008) Isolation of the cDNAs encoding secreted and membrane binding proteins from egg of Schistosoma japonicum (Chinese strain). Acta Parasitologica 1: 110–114.

[27] Mbanefo EC, Yu C, Kikuchi M, Shuaibu NM, Boamah D, et al (2012) Origin of a novel protein-coding gene family with similar signal sequence in Schistosoma japonicum. BMC Genomics 13: 260. 22716200 10.1186/1471-2164-13-260 PMC3434034

[28] Bork P, Patthy L (1995) The SEA module: a new extracellular domain associated with O-glycosylation. Protein Sci 4: 1421–1425. 7670383 10.1002/pro.5560040716 PMC2143162

[29] Maeda T, Inoue M, Koshiba S, Yabuki T, Aoki M, et al (2004) Solution structure of the SEA domain from the murine homologue of ovarian cancer antigen CA125 (MUC16). J Biol Chem 279: 13174–13182. 14764598 10.1074/jbc.M309417200

[30] Palmai-Pallag T, Khodabukus N, Kinarsky L, Leir SH, Sherman S, et al (2005) The role of the SEA (sea urchin sperm protein, enterokinase and agrin) module in cleavage of membrane-tethered mucins. FEBS J 272: 2901–2911. 15943821 10.1111/j.1742-4658.2005.04711.x

[31] Corpet F (1988) Multiple sequence alignment with hierarchical clustering. Nucleic Acids Res 16: 10881–10890. 2849754 10.1093/nar/16.22.10881 PMC338945

[32] Gupta R, Brunak S (2002) Prediction of glycosylation across the human proteome and the correlation to protein function. Pac Symp Biocomput 310–322. 11928486

[33] Kelley LA, Sternberg MJ (2009) Protein structure prediction on the Web: a case study using the Phyre server. Nat Protoc 4: 363–371. 19247286 10.1038/nprot.2009.2

[34] Raman S, Vernon R, Thompson J, Tyka M, Sadreyev R, et al (2009) Structure prediction for CASP8 with all-atom refinement using Rosetta. Proteins 77 (Suppl 9) 89–99. 19701941 10.1002/prot.22540 PMC3688471

[35] Wass MN, Kelley LA, Sternberg MJ (2010) 3DLigandSite: predicting ligand-binding sites using similar structures. Nucleic Acids Res 38: W469–473. 20513649 10.1093/nar/gkq406 PMC2896164

[36] Tansatit T, Sahaphong S, Riengrojpitak S, Viyanant V, Sobhon P (2006) Immunolocalization of cytoskeletal components in the tegument of the 3-week-old juvenile and adult Fasciola gigantica. Vet Parasitol 135: 269–278. 16310956 10.1016/j.vetpar.2005.10.018

[37] Suda Y, Arano A, Fukui Y, Koshida S, Wakao M, et al (2006) Immobilization and clustering of structurally defined oligosaccharides for sugar chips: an improved method for surface plasmon resonance analysis of protein-carbohydrate interactions. Bioconjug Chem 17: 1125–1135. 16984119 10.1021/bc0600620

[38] Asuthkar S, Velineni S, Stadlmann J, Altmann F, Sritharan M (2007) Expression and characterization of an iron-regulated hemin-binding protein, HbpA, from Leptospira interrogans serovar Lai. Infect Immun 75: 4582–4591. 17576761 10.1128/IAI.00324-07 PMC1951163

[39] Huy NT, Xuan Trang DT, Uyen DT, Sasai M, Harada S, et al (2005) An improved colorimetric method for quantitation of heme using tetramethylbenzidine as substrate. Anal Biochem 344: 289–291. 16091279 10.1016/j.ab.2005.06.022

[40] Huy NT, Kamei K, Yamamoto T, Kondo Y, Kanaori K, et al (2002) Clotrimazole binds to heme and enhances heme-dependent hemolysis: proposed antimalarial mechanism of clotrimazole. J Biol Chem 277: 4152–4158. 11707446 10.1074/jbc.M107285200

[41] Akhavan A, Crivelli SN, Singh M, Lingappa VR, Muschler JL (2008) SEA domain proteolysis determines the functional composition of dystroglycan. FASEB J 22: 612–621. 17905726 10.1096/fj.07-8354com

[42] Levitin F, Stern O, Weiss M, Gil-Henn C, Ziv R, et al (2005) The MUC1 SEA module is a self-cleaving domain. J Biol Chem 280: 33374–33386. 15987679 10.1074/jbc.M506047200

[43] Huy NT, Serada S, Trang DT, Takano R, Kondo Y, et al (2003) Neutralization of toxic heme by Plasmodium falciparum histidine-rich protein 2. J Biochem 133: 693–698. 12801923 10.1093/jb/mvg089

[44] Lin X (2004) Functions of heparan sulfate proteoglycans in cell signaling during development. Development 131: 6009–6021. 15563523 10.1242/dev.01522

[45] Tumova S, Woods A, Couchman JR (2000) Heparan sulfate proteoglycans on the cell surface: versatile coordinators of cellular functions. Int J Biochem Cell Biol 32: 269–288. 10716625 10.1016/s1357-2725(99)00116-8

[46] Derksen PW, Keehnen RM, Evers LM, van Oers MH, Spaargaren M, et al (2002) Cell surface proteoglycan syndecan-1 mediates hepatocyte growth factor binding and promotes Met signaling in multiple myeloma. Blood 99: 1405–1410. 11830493 10.1182/blood.v99.4.1405

[47] Chua CC, Rahimi N, Forsten-Williams K, Nugent MA (2004) Heparan sulfate proteoglycans function as receptors for fibroblast growth factor-2 activation of extracellular signal-regulated kinases 1 and 2. Circ Res 94: 316–323. 14684627 10.1161/01.RES.0000112965.70691.AC

[48] Couchman JR (2010) Transmembrane signaling proteoglycans. Annu Rev Cell Dev Biol 26: 89–114. 20565253 10.1146/annurev-cellbio-100109-104126

[49] Nam EJ, Park PW (2012) Shedding of cell membrane-bound proteoglycans. Methods Mol Biol 836: 291–305. 22252642 10.1007/978-1-61779-498-8_19 PMC3569011

[50] Wegrowski Y, Milard AL, Kotlarz G, Toulmonde E, Maquart FX, et al (2006) Cell surface proteoglycan expression during maturation of human monocytes-derived dendritic cells and macrophages. Clin Exp Immunol 144: 485–493. 16734618 10.1111/j.1365-2249.2006.03059.x PMC1941969

[51] van Die I, Cummings RD (2010) Glycan gimmickry by parasitic helminths: a strategy for modulating the host immune response? Glycobiology 20: 2–12. 19748975 10.1093/glycob/cwp140

[52] Sook BR, Block DR, Sumithran S, Montañez GE, Rodgers KR, et al (2008) Characterization of SiaA, a streptococcal heme-binding protein associated with a heme ABC transport system. Biochemistry 47: 2678–2688. 18247478 10.1021/bi701604y

[53] Cupello MP, Souza CF, Buchensky C, Soares JB, Laranja GA, et al (2011) The heme uptake process in Trypanosoma cruzi epimastigotes is inhibited by heme analogues and by inhibitors of ABC transporters. Acta Trop 120: 211–218. 21903090 10.1016/j.actatropica.2011.08.011

[54] Walker AJ (2011) Insights into the functional biology of schistosomes. Parasit Vectors 4: 203. 22013990 10.1186/1756-3305-4-203 PMC3206467

[55] Wu XJ, Sabat G, Brown JF, Zhang M, Taft A, et al (2009) Proteomic analysis of Schistosoma mansoni proteins released during in vitro miracidium-to-sporocyst transformation. Mol Biochem Parasitol 164: 32–44. 19095013 10.1016/j.molbiopara.2008.11.005 PMC2665799

[56] Pascoa V, Oliveira PL, Dansa-Petretski M, Silva JR, Alvarenga PH, et al (2002) Aedes aegypti peritrophic matrix and its interaction with heme during blood digestion. Insect Biochem Mol Biol 32: 517–523. 11891128 10.1016/s0965-1748(01)00130-8

[57] Devenport M, Alvarenga PH, Shao L, Fujioka H, Bianconi ML, et al (2006) Identification of the Aedes aegypti peritrophic matrix protein AeIMUCI as a heme-binding protein. Biochemistry 45: 9540–9549. 16878988 10.1021/bi0605991

