# Supplementary figures and images for: Characterization of a Gene Family Encoding SEA (Sea-urchin Sperm Protein, Enterokinase and Agrin)-Domain Proteins with Lectin-Like and Heme-Binding Properties from Schistosoma japonicum
Source: PLoS Negl Trop Dis. 2014 Jan 9;8(1):e2644. doi: 10.1371/journal.pntd.0002644 (PMC3886910; doi:10.1371/journal.pntd.0002644)

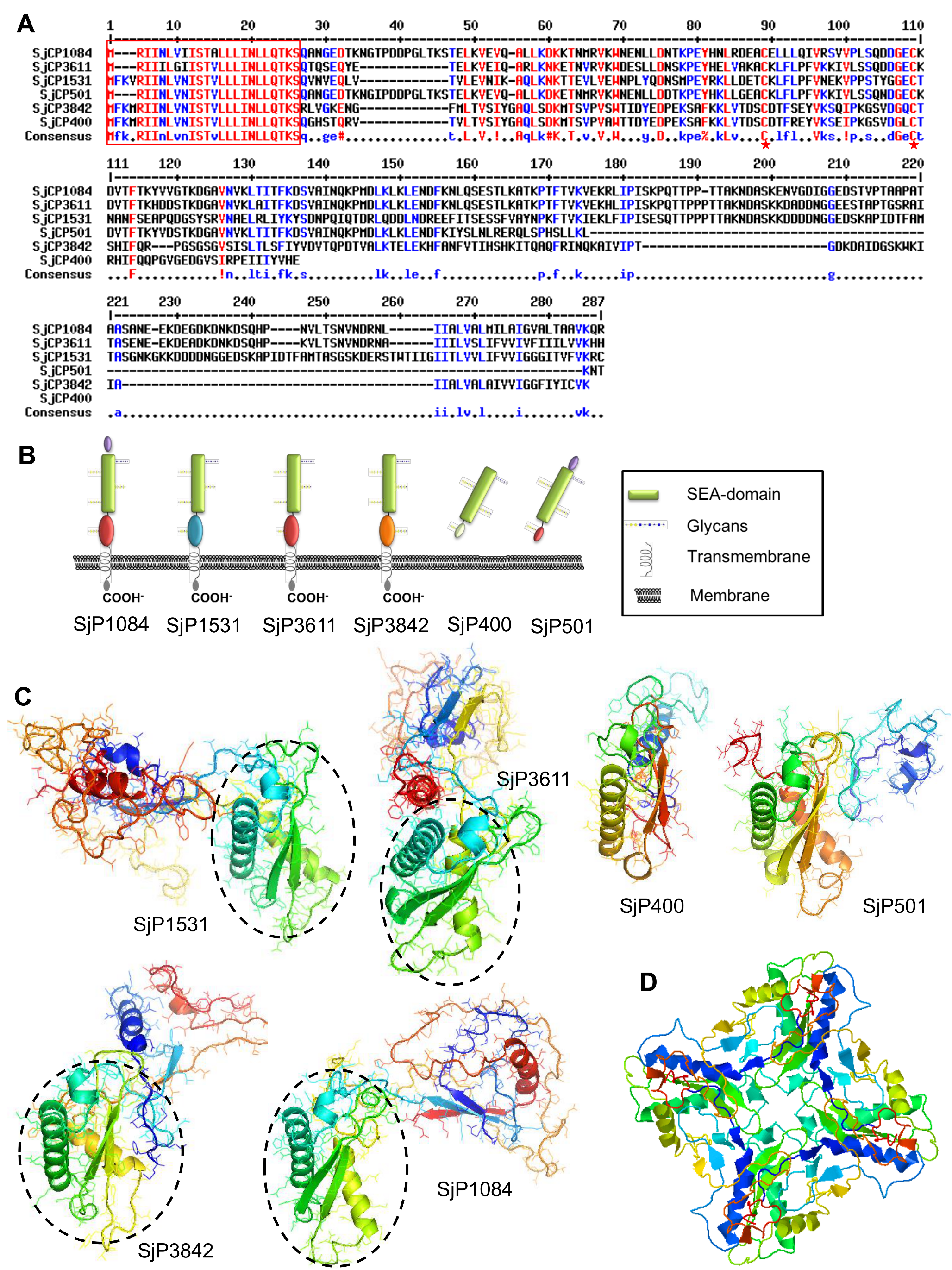

Supplement: Figure S1 — Multiple alignments and molecular structures of the SEA-domain proteins from S. japonicum . (A) Multiple alignments of SST isolated candidates showing similar signal sequence [27]. (B) The Topology of the members of the gene family. (C) The modeled molecular structures of whole molecules showing the SEA-domain. (D) Predicted oligomeric state of SjCP3842 as a representative of the family. (TIF) [file pntd.0002644.s001.tif]

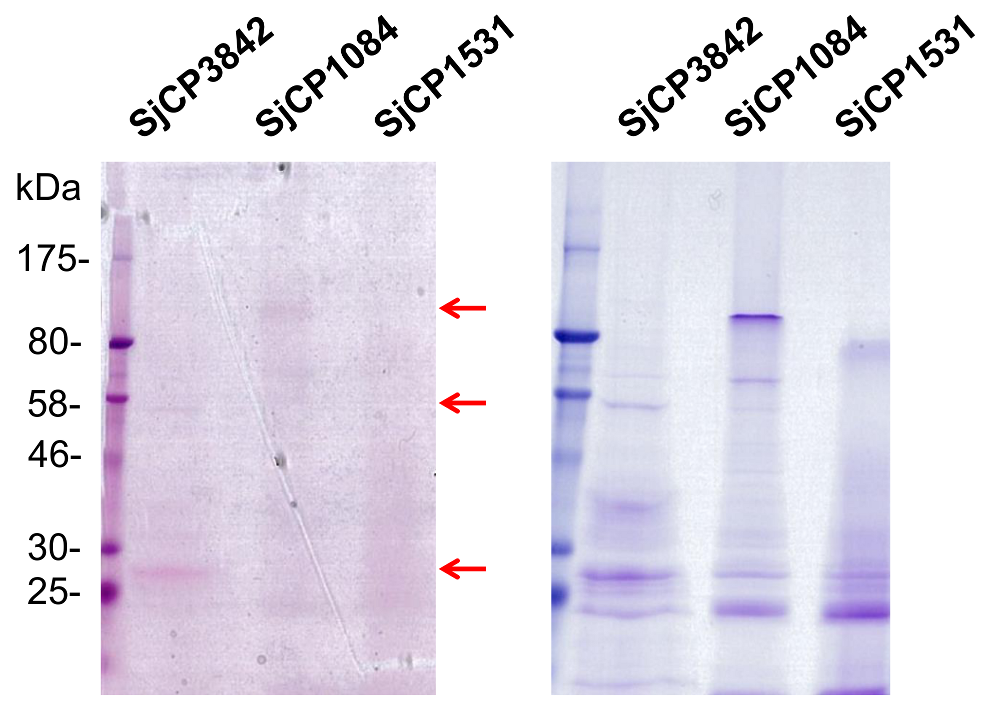

Supplement: Figure S2 — Glycoprotein detection analysis showed evidence of O -linked glycosylation in the expressed candidate proteins. We showed using glycosylation detection assay that the expressed proteins contain O-linked glycans. Glycosylated proteins were detected as magenta stained bands in SDS-PAGE fractions. (TIF) [file pntd.0002644.s002.tif]

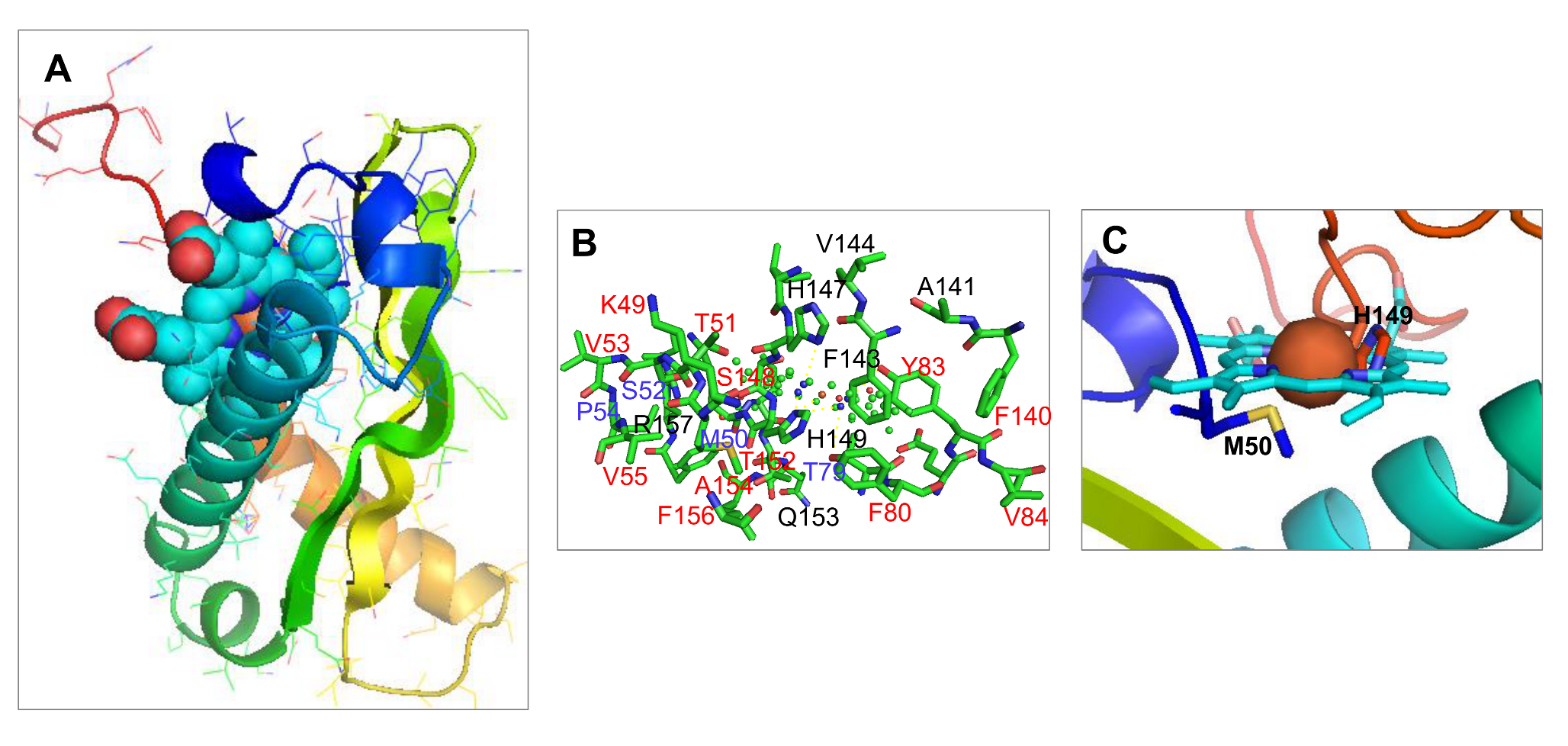

Supplement: Figure S4 — Heme-binding pocket of SjCP3842. (A) Heme-binding mode of SjCP3842 showing the hydrophobic vinyl end of heme inserted into a hydrophobic cavity, while hydrophilic propionate end points away from the pocket. Heme is represented using spheres model colored by atoms (C: green, N: blue, O: red, Fe: brown). (B) The interacting residues in the heme-binding site. Strictly conserved residues are labeled in red while partly conserved residues are labeled in blue. Heme molecule is shown with nb-spheres model while residues are shown with sticks model, both colored by atoms. (C) The second predicted heme-binding mode showing heme iron hexa-coordinated with His-149 and Met-50 as axial ligands. (TIF) [file pntd.0002644.s004.tif]

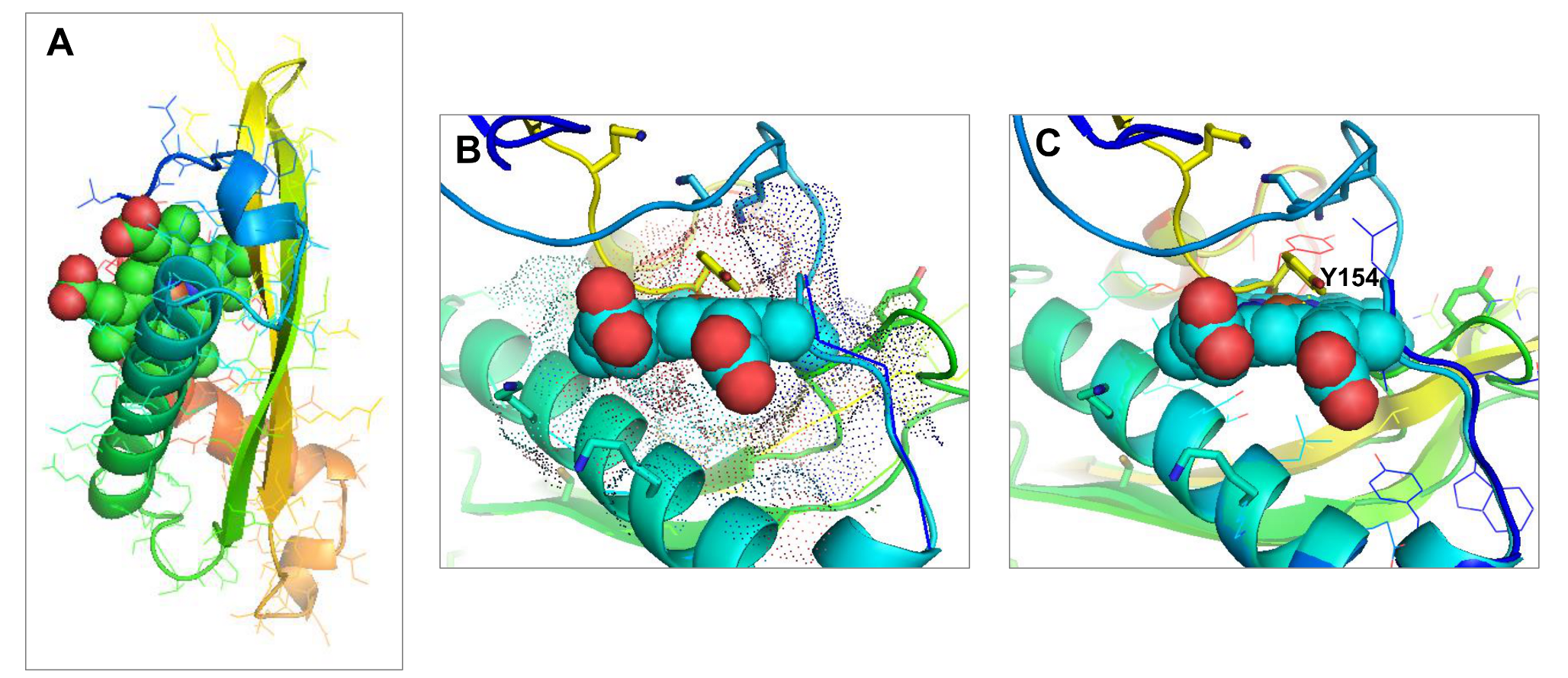

Supplement: Figure S5 — Heme binding pocket of SjCP1531. Similar binding site characteristics as observed for SjCP3842 were observed for SjCP1531. However, heme iron is coordinated to Tyr-154 as its axial ligand. (A) Heme-binding mode of SjCP1531 showing the hydrophobic vinyl end of the protoporphyrin heme inserted into a hydrophobic cavity, while hydrophilic propionate ends of points away from the pocket. Heme is represented using spheres model colored by atoms (C: green, N: blue, O: red, Fe: brown). The protein is shown using cartoon model. (B) Heme-binding site showing the Connolly surface of the binding pocket (dots). (C) Heme iron (brown sphere) putatively coordinated to Tyr-154 as axial ligand. (TIF) [file pntd.0002644.s005.tif]

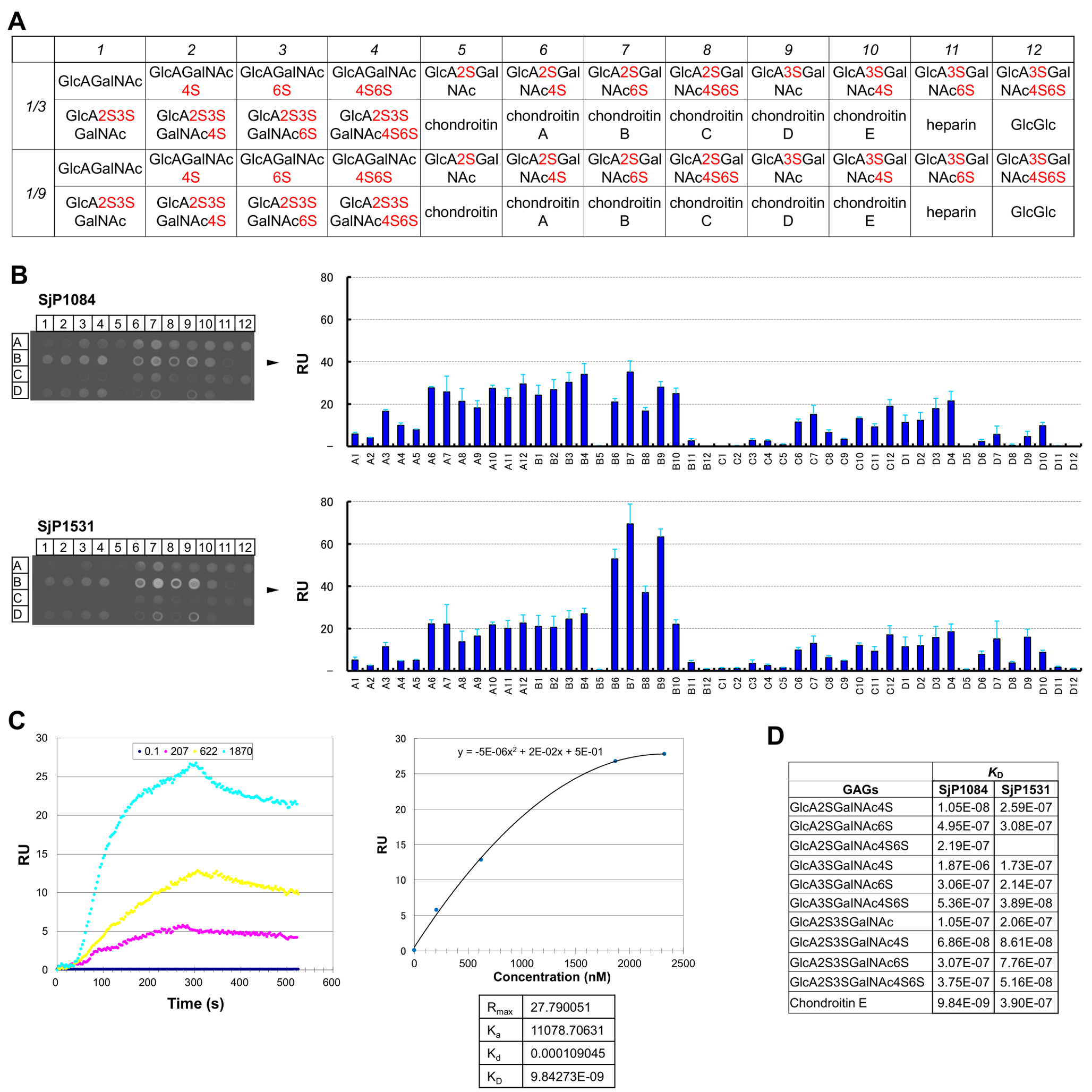

Supplement: Figure S6 — Specificity and affinity of protein-glycan interactions. (A) Array format of CS-GAG chip. (B) SPR imaging of the glycan-binding assay are shown. (C) Sensorgram and binding curve of the interaction between SjCP1084 and chondroitin sulfate-E as representative of the binding kinetics data. (D) Summary of equilibrium dissociation constants (K D) of protein-glycan interactions, showing values within nanomolar range. (TIF) [file pntd.0002644.s006.tif]

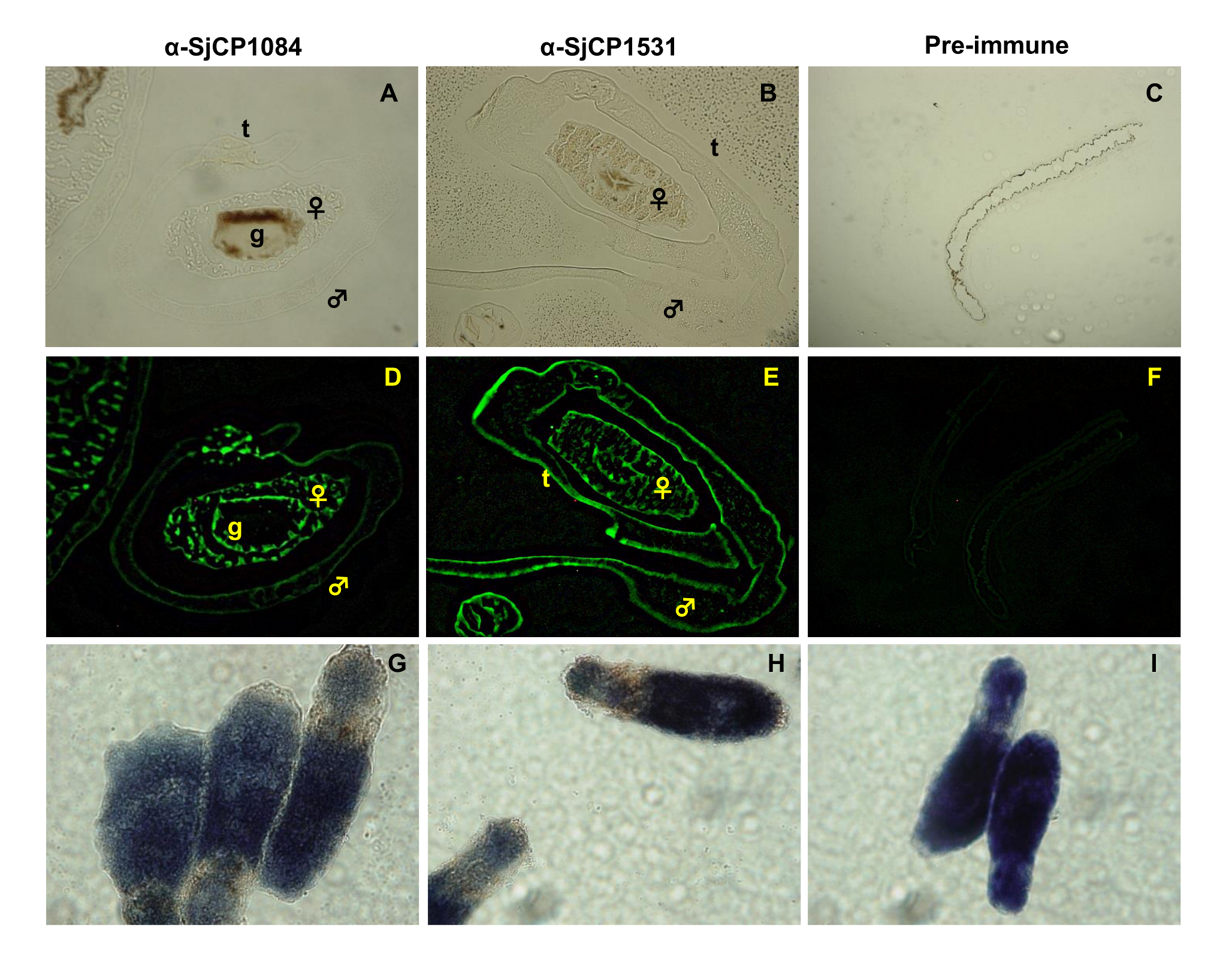

Supplement: Figure S7 — Immunolocalization on the teguments and gut epithelial linings. Immunolocalization of SjCP1084 (A and D) and SjCP1531 (B and E) using IFA on cross sections of adult worm pairs probed with immune sera and detected with FITC conjugated secondary antibody. Immunoperoxidase detection of SjCP1084 (G) and SjCP1531 (H) on the juvenile schistosomulae using immune sera and HRP conjugated secondary antibodies also showed localization on the tegument. No signal was detected in adult worm sections and schistosomulae probed with the pre-immune serum (C, F and I). (TIF) [file pntd.0002644.s007.tif]
