# Supplementary material for: Characterization of a Gene Family Encoding SEA (Sea-urchin Sperm Protein, Enterokinase and Agrin)-Domain Proteins with Lectin-Like and Heme-Binding Properties from Schistosoma japonicum
Source: PLoS Negl Trop Dis. 2014 Jan 9;8(1):e2644. doi: 10.1371/journal.pntd.0002644 (PMC3886910; doi:10.1371/journal.pntd.0002644)
Supplement: Figure S3 — Molecular structure based identification of heme-binding site. 3DLigandSite ligand binding site prediction report showing structure based identification of heme-binding site at highly significant precision. The heme-binding site was predicted based on 178 ligands present in 177 homologous structures. (PDF) [file pntd.0002644.s003.pdf]

Imperial College London

Structural Bioinformatics Group

Division of Molecular Biosciences
Faculty of Natural Sciences

3DLigandSite -Ligand binding site prediction Server

Home About News Help FAQ Example Contact Disclaimer

3DLigandSite Results

Submission Successful - Job-id: 68c91ad1f31a21fe Current Status: STATUS:finished

All Jobs are run on a queue system. You will receive an email confirming submission and one once 3dLigandSite has run. Jobs generally take between 1-3 hours to complete depending on complexity and server load.
you can also view your job's progress and results on this page It will refresh every 30 second until the job is completed
You can bookmark this page: http://www.sbg.bio.imperial.ac.uk/3dligandsite/3dligand_report_p2.cgi?jobid=68c91ad1f31a21fe

Please cite: Wass M.N., Kelley L.A. and Sternberg M.J. (2010) 3DLigandSite: predicting ligand-binding sites using similar structures.*NAR* 38, W469-73. PubMed

The structural library currently used by 3DLigandSite is based on the PDB as of 20 January 2010

Submission Details

**Email:**	evari4u@gmail.com
**Unique Job identifier:**	68c91ad1f31a21fe
**Description:**	SjP3842 repeat
**Date:**	Tue Jun 18 10:26:14 BST 2013
**Submission Type:**	structure
**Query Seq:**	KMTSVPVSWTIDYEDPEKSAFKKLVTDSCDTFSEYVKSQIPKGSVDGQCTSHIFQRPGSG<br>SGVSISLTLSFIYVDVTQPDTVALKTELEKHFANFVTIHSHKITQAQFRIN

Structural Model

**User provided file:**	SjP3842.pdb

Structural Search

confidence data from search of structural library with Mammoth

**Average lnE:**	7.40243
**Maximum LnE:**	8.11
**Min LnE:**	7.075

Ligand Clusters Identified

Note prediction based on first cluster
Click on other clusters to view the potential sites associated with them

			MAMMOTH Scores		
**Cluster**	**Ligands**	**Structures**	**Av**	**min**	**max**
1	178	177	7.4	7.1	8.1
2	3	2	8.2	7.2	9.2
3	2	2	7.1	7.1	7.1
4	2	2	7.1	7.1	7.1
7	1	1	7.4	7.4	7.4
5	1	1	7.2	7.2	7.2
6	1	1	7.2	7.2	7.2

Predicted Binding Site

Residue	Amino acid	contact	av distance	JS divergence
49	LYS	177	0.00	0.64
50	MET	178	0.00	0.42
51	THR	177	0.00	0.62
52	SER	171	0.00	0.55
54	PRO	176	0.00	0.55
55	VAL	176	0.00	0.69
75	ASP	109	0.27	0.58

Heterogens present in Predicted Binding Site

Heterogen	Count	source structures
HEC	1	2h35_B
HEM	176	1pbx_B,1cg5_B,3bj1_D,1y4r_B,1y0c_B,<br>1xzv_B,1xzu_B,1xy0_B,1xq5_B,1x46_A,<br>1nqp_B,1hv4_B,1c40_B,2hhe_B,2dn1_B,<br>1yvq_B,1y85_B,1y4g_B,1v4x_D,1v4w_B,<br>1spg_A,1rvw_B,1qi8_D,1ouu_D,1o1p_B,<br>1lfl_Q,1j7s_D,1ibe_B,1dke_D,1ch4_D,<br>1bij_B,1a9w_E,6hbw_D,3k9z_A,3fs4_B,<br>3d7o_B,3bj2_B,2r80_B,2mhb_B,2dxm_B,<br>2dn3_B,2dn2_D,1yvt_B,1yff_F,1ye1_B,<br>1y7c_B,1y4q_B,1y4p_B,1y45_B,1y0t_B,<br>1xye_B,1xxt_D,1sdl_B,1r1y_B,1r1x_B,

Print to PDF without this message by purchasing novaPDF (http://www.novapdf.com/)

Residue	Amino acid	contact	av distance	JS divergence
79	THR	177	0.00	0.61
80	PHE	167	0.02	0.70
83	TYR	114	0.28	0.74
111	VAL	134	0.05	0.78
112	SER	50	0.45	0.63
143	PHE	158	0.22	0.53
149	HIS	45	0.25	0.55
150	LYS	140	0.32	0.56
151	ILE	177	0.00	0.59
153	GLN	174	0.13	0.40

		1mko_B,1k0y_D,1ird_B,1hds_D,1hbb_B, 1gcv_C,1g9v_B,1g08_B,1cg8_B,1c7b_B, 1bz1_D,1bz0_D,1bbb_D,1abw_D,1a0u_B, 3dhr_H,2zwj_A,2mge_A,2hbe_B,2evk_A, 2d5x_B,1yie_D,1ygf_B,1yg5_D,1x3k_A, 1uiw_D,1spg_B,1mnj_B,1ljw_B,1j41_F, 1iwh_B,1i3d_A,1g0a_D,1ecn_A,1a4f_B, 3d17_C,2z6n_B,2mye_A,2mgd_A,2hhb_B, 2g14_A,2g12_A,2g11_A,2g10_A,2g0s_A, 2d60_D,1ymb_A,1yen_D,1ydz_B,1wmu_B, 1vxb_A,1urv_A,1ns6_A,1mym_A,1mni_A, 1mlr_A,1m9p_D,1j7y_B,1irc_A,1hbr_B, 1gcw_A,1gbv_B,1gbu_B,1eco_A,1ecd_A, 1eca_A,1duk_A,1dtm_A,1ch9_A,1buw_D, 1a6g_A,1a3n_D,108m_A,4mbn_A,3ecz_A, 2z6t_A,2z6s_A,2peg_B,2nsr_A,2mya_A, 2mgj_A,2hhd_B,2g0z_A,2g0x_A,2g0v_A, 2g0r_A,2evp_A,1ygd_D,1y0a_D,1xz5_D, 1xch_A,1wla_A,1vxa_A,1o1o_B,1o1n_B, 1nz2_A,1myg_B,1mnk_B,1mlm_A,1j7w_B, 1j40_D,1ith_A,1hdb_B,1dxv_B,1dxu_B, 1dxt_B,1dwt_A,1duo_A,1dti_A,1cls_B, 1c7d_D,1bzz_B,1bab_B,1azi_A,1ajh_A, 1ajg_A,1abs_A,1a6n_A,1a6m_A,1a3o_B, 112m_A
FE2	1	3k9z_A

Structural View of Prediction

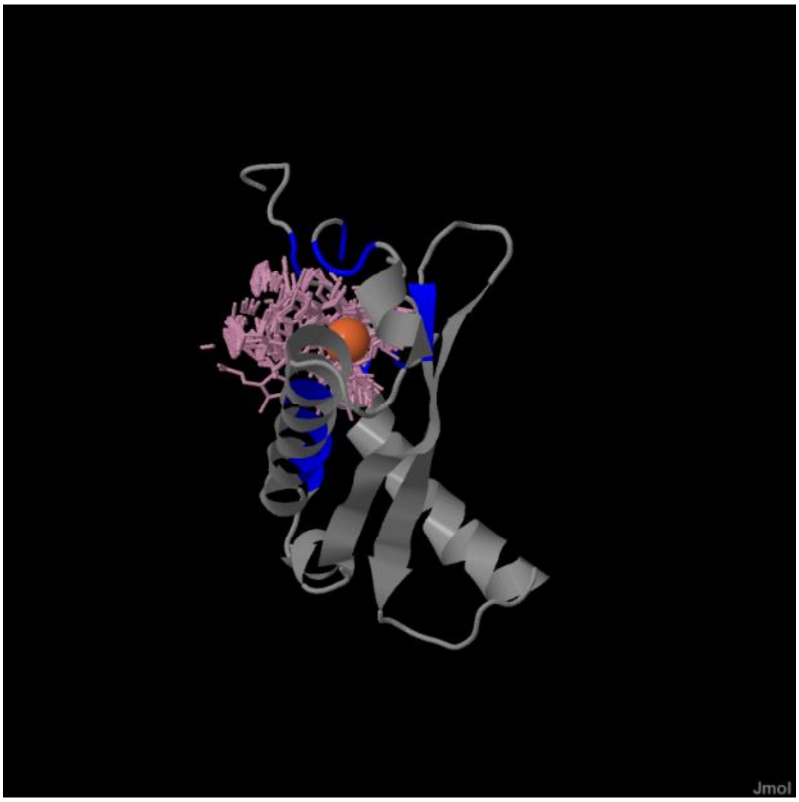

Take snapshot of current view

Download model and pymol Script

Display Modification

Whole protein

colour by: ◉ prediction ○ Jensen Shannon Divergence

spacefill: ◉ off ○ 20% ○ 100%

wireframe: ◉ off ○ wireframe ○ wireframe 50 ○ wireframe 100

☑ cartoon

Predicted residues

spacefill: ◉ off ○ 20% ○ 100%

wireframe: ◉ off ○ on ○ wireframe 50 ○ wireframe 100

☑ cartoon
☐ label

Heterogens

Display of Metalic heterogens
spacefill: ○ off ○ 20% ◉ 100%

Display of Non Metalic heterogens
spacefill: ◉ off ○ 20% ○ 100%

wireframe: ○ off ○ standard ◉ wireframe 50 ○ wireframe 100

View

Reset to original orientation

☐ spin

background black

Prediction colour legend:	Other residues	Predicted Binding Site		
Conservation Score Colour legend:	0-0.15	0.16-0.30	0.31-0.40	0.41-0.50
	0.51-0.60	0.61-0.70	0.71-0.80	0.81-1.00

Home About News Help FAQ Example Contact Disclaimer

© Structural Bioinformatics Group, Imperial College, London
Mark Wass
