# Supplementary material for: Characterization of a Gene Family Encoding SEA (Sea-urchin Sperm Protein, Enterokinase and Agrin)-Domain Proteins with Lectin-Like and Heme-Binding Properties from Schistosoma japonicum
Source: PLoS Negl Trop Dis. 2014 Jan 9;8(1):e2644. doi: 10.1371/journal.pntd.0002644 (PMC3886910; doi:10.1371/journal.pntd.0002644)
Supplement: Table S1 — Summary of structural homology modeling results for S. japonicum SEA-domain gene family. In addition to the structural homology modeling data presented in Figure 1, the structural modeling was equally performed for all identified transcripts in this gene family and the result is summarized in this table. (DOCX) [file pntd.0002644.s008.docx]

**Table S1. Summary of structural homology modeling results for *S. japonicum* SEA-domain gene family**

| **UniGene (UID)** | **Transcripts (GenBank)** | **GenBank Annotation** | **Top Models** | **Confid.* (%)** |
| --- | --- | --- | --- | --- |
| Sja.1676 (1476312) | AY570748^SST^ | Egg protein SjCP3842 | SEA-domain of TMPRSS2 [PDB: 2E7V] | 96.3 |
|  | AY223245 | Hypothetical protein | SEA-domain of TMPRSS2 [PDB: 2E7V] | 96 |
|  | AY222916 | Hypothetical protein | SEA-domain of TMPRSS2 [PDB: 2E7V] | 95 |
|  | AY813542 | Egg protein CP3842 | SEA-domain of TMPRSS2 [PDB: 2E7V] | 93.9 |
|  | EF127834 | Unknown | SEA-domain of TMPRSS2 [PDB: 2E7V] | 96.1 |
|  | EF140742 | Somula protein | SEA-domain of TMPRSS2 [PDB: 2E7V] | 95.8 |
|  | FN323799 | Egg protein CP3842 | SEA-domain of TMPRSS2 [PDB: 2E7V] | 96.3 |
|  | FN323800 | Egg protein CP3842 | SEA-domain of TMPRSS2 [PDB: 2E7V] | 95.9 |
|  | FN323801 | Egg protein CP3842 | SEA-domain of TMPRSS2 [PDB: 2E7V] | 96.2 |
|  | FN323803 | Somula protein | SEA-domain of TMPRSS2 [PDB: 2E7V] | 96.2 |
|  | FN323793 | Egg protein CP3842 | SEA-domain of TMPRSS2 [PDB: 2E7V] | 96.3 |
|  | FN323792 | Egg protein CP3842 | SEA-domain of TMPRSS2 [PDB: 2E7V] | 96.3 |
|  | FN323791 | Egg protein CP3842 | SEA-domain of TMPRSS2 [PDB: 2E7V] | 96.3 |
|  | FN323790 | Egg protein CP3842 | SEA-domain of TMPRSS2 [PDB: 2E7V] | 96.3 |
|  | FN323788 | Egg protein CP3842 | SEA-domain of TMPRSS2 [PDB: 2E7V] | 96.3 |
|  | FN323785 | Egg protein CP3842 | SEA-domain of TMPRSS2 [PDB: 2E7V] | 96.3 |
|  | FN323782 | Egg protein CP3842 | SEA-domain of TMPRSS2 [PDB: 2E7V] | 96.3 |
|  | FN323781 | Egg protein CP3842 | SEA-domain of TMPRSS2 [PDB: 2E7V] | 96.3 |
|  | FN323779 | Egg protein CP3842 | SEA-domain of TMPRSS2 [PDB: 2E7V] | 96.3 |
|  | FN323778 | Hypothetical protein | SEA-domain of TMPRSS2 [PDB: 2E7V] | 96.3 |
|  | FN323777 | Somula protein | SEA-domain of TMPRSS2 [PDB: 2E7V] | 96.2 |
|  | FN323776 | Egg protein CP3842 | SEA-domain of TMPRSS2 [PDB: 2E7V] | 96.3 |
|  | FN323773 | Egg protein CP3842 | SEA-domain of TMPRSS2 [PDB: 2E7V] | 96.3 |
|  | FN323772 | Egg protein CP3842 | SEA-domain of TMPRSS2 [PDB: 2E7V] | 96.3 |
|  | FN323771 | Egg protein CP3842 | SEA-domain of TMPRSS2 [PDB: 2E7V] | 96.3 |
|  | FN323770 | Egg protein CP3842 | SEA-domain of TMPRSS2 [PDB: 2E7V] | 96.3 |
|  | FN323769 | Egg protein CP3842 | SEA-domain of TMPRSS2 [PDB: 2E7V] | 95.9 |
|  | FN323768 | Egg protein CP3842 | SEA-domain of TMPRSS2 [PDB: 2E7V] | 96.3 |
|  | FN323767 | Egg protein CP3842 | SEA-domain of TMPRSS2 [PDB: 2E7V] | 96.3 |
|  | FN323766 | Egg protein CP3842 | SEA-domain of TMPRSS2 [PDB: 2E7V] | 95.9 |
|  | FN323765 | Egg protein CP3842 | SEA-domain of TMPRSS2 [PDB: 2E7V] | 96.3 |
|  | FN323764 | Somula protein | SEA-domain of TMPRSS2 [PDB: 2E7V] | 96.2 |
|  | FN323763 | Egg protein CP3842 | Sh3-domain of obscurin [PDB: 1V1C] | 82.4 |
|  | FN323762 | Egg protein CP3842 | SEA-domain of TMPRSS2 [PDB: 2E7V] | 96.3 |
|  | BU772060^EST^ | Unknown | N/A | N/A |
|  | BU766145^EST^ | Unknown | N/A | N/A |
|  | CX862012^EST^ | Unknown | N/A | N/A |
| Sja.13324 (3987052) | AY570737^SST^ | Egg protein SjCP1084 | SEA-domain of TMPRSS2 [PDB: 2E7V] | 93.7 |
|  | FN328299^NC^ | Non-coding | N/A | N/A |
| Sja.11891 (2895889) | AY813975 | Egg protein CP1084 | SEA-domain of TMPRSS2 [PDB: 2E7V] | 92.4 |
|  | FN329814^NC^ | Non-coding | N/A | N/A |
|  | BU769048^EST^ | Unknown | N/A | N/A |
| Sja.14562 (3988290) | FN327130 | Egg protein CP1084 | SEA-domain of TMPRSS2 [PDB: 2E7V] | 95.3 |
|  | FN326955 | Egg protein CP1084 | SEA-domain of TMPRSS2 [PDB: 2E7V] | 95.3 |
|  | FN326901 | Egg protein CP1084 | SEA-domain of TMPRSS2 [PDB: 2E7V] | 95.3 |
| Sja.14565 (3988293) | FN327099 | Egg protein CP1084 | SEA-domain of TMPRSS2 [PDB: 2E7V] | 89.7 |
| Sja.2063 (1476798) | FN321064 | Egg protein CP1084 | SEA-domain of TMPRSS2 [PDB: 2E7V] | 94 |
|  | FN321061 | Egg protein CP1084 | SEA-domain of TMPRSS2 [PDB: 2E7V] | 94 |
| Sja.2065 (1476800) | AY570753^SST^ | Egg protein SjCP501 | SEA-domain of TMPRSS2 [PDB: 2E7V] | 92.4 |
|  | AY570744^SST^ | Egg protein SjCP3611 | SEA-domain of TMPRSS2 [PDB: 2E7V] | 90.9 |
|  | AY814685 | Egg protein CP3611 | SEA-domain of TMPRSS2 [PDB: 2E7V] | 91 |
|  | FN327232 | Hypothetical protein | SEA-domain of TMPRSS2 [PDB: 2E7V] | 92.3 |
|  | FN327137 | Hypothetical protein | SEA-domain of TMPRSS2 [PDB: 2E7V] | 92.3 |
|  | FN318042 | Hypothetical protein | SEA-domain of TMPRSS2 [PDB: 2E7V] | 95.2 |
|  | FN321065 | Egg protein CP3611 | SEA-domain of TMPRSS2 [PDB: 2E7V] | 90.9 |
|  | FN321060 | Hypothetical protein | SEA-domain of TMPRSS2 [PDB: 2E7V] | 93.9 |
|  | FN321059 | Hypothetical protein | SEA-domain of TMPRSS2 [PDB: 2E7V] | 90.6 |
|  | FN321058 | Hypothetical protein | SEA-domain of TMPRSS2 [PDB: 2E7V] | 91.9 |
|  | FN321057 | Hypothetical protein | SEA-domain of TMPRSS2 [PDB: 2E7V] | 90.6 |
|  | FN321056 | Hypothetical protein | SEA-domain of TMPRSS2 [PDB: 2E7V] | 91.6 |
|  | FN321055 | Hypothetical protein | SEA-domain of TMPRSS2 [PDB: 2E7V] | 91.6 |
|  | FN329815^NC^ | Non-coding | N/A | N/A |
|  | BU768978^EST^ | Unknown | N/A | N/A |
|  | BU780021^EST^ | Unknown | N/A | N/A |
| Sja.1526 (1476162) | AY814448 | Egg protein CP3611 | SEA-domain of TMPRSS2 [PDB: 2E7V] | 94 |
|  | BU780442^EST^ | Unknown | N/A | N/A |
| Sja.11083 (2671933) | AY915467 | Hypothetical protein | Photosystem II (PsbM-like) [SCOP: 2AXT] | 48.2 |
|  | FN327219 | Hypothetical protein | SEA-domain of TMPRSS2 [PDB: 2E7V] | 94.6 |
|  | FN327063 | Hypothetical protein | SEA-domain of TMPRSS2 [PDB: 2E7V] | 94.2 |
|  | FN326828 | Hypothetical protein | SEA-domain of TMPRSS2 [PDB: 2E7V] | 94 |
|  | FN326826 | Somula protein | SEA-domain of TMPRSS2 [PDB: 2E7V] | 96.1 |
|  | FN323794 | Hypothetical protein | SEA-domain of TMPRSS2 [PDB: 2E7V] | 94.1 |
|  | FN323797 | Hypothetical protein | SEA-domain of TMPRSS2 [PDB: 2E7V] | 94.1 |
|  | FN323798 | Hypothetical protein | SEA-domain of TMPRSS2 [PDB: 2E7V] | 95.3 |
|  | FN323802 | Hypothetical protein | SEA-domain of TMPRSS2 [PDB: 2E7V] | 94.2 |
|  | FN323789 | Hypothetical protein | SEA-domain of TMPRSS2 [PDB: 2E7V] | 94.2 |
|  | FN323787 | Hypothetical protein | SEA-domain of TMPRSS2 [PDB: 2E7V] | 94.1 |
|  | FN323786 | Hypothetical protein | SEA-domain of TMPRSS2 [PDB: 2E7V] | 94.2 |
|  | FN323784 | Hypothetical protein | SEA-domain of TMPRSS2 [PDB: 2E7V] | 94.2 |
|  | FN323783 | Hypothetical protein | SEA-domain of TMPRSS2 [PDB: 2E7V] | 94.1 |
|  | FN323780 | Hypothetical protein | SEA-domain of TMPRSS2 [PDB: 2E7V] | 95.4 |
|  | FN323774 | Hypothetical protein | SEA-domain of TMPRSS2 [PDB: 2E7V] | 95.5 |
|  | FN323761 | Hypothetical protein | Pro-sfti-1 [PDB: 2AB9] | 20.3 |
|  | FN323760 | Hypothetical protein | SEA-domain of TMPRSS2 [PDB: 2E7V] | 90.3 |
|  | FN323759 | Hypothetical protein | SEA-domain of TMPRSS2 [PDB: 2E7V] | 90.3 |
|  | FN323758 | Hypothetical protein | SEA-domain of TMPRSS2 [PDB: 2E7V] | 95.2 |
|  | FN323757 | Hypothetical protein | SEA-domain of TMPRSS2 [PDB: 2E7V] | 94.2 |
|  | FN320521 | Hypothetical protein | SEA-domain of TMPRSS2 [PDB: 2E7V] | 88.7 |
|  | FN320520 | Hypothetical protein | SEA-domain of MUC16 [PDB: 1IVZ] | 94.2 |
|  | FN320519 | Hypothetical protein | PsbM-like [SCOP: 2AXT] | 55.4 |
|  | FN320518 | Hypothetical protein | SEA-domain of TMPRSS2 [PDB: 2E7V] | 95.5 |
|  | FN320517 | Hypothetical protein | SEA-domain of TMPRSS2 [PDB: 2E7V] | 93.6 |
|  | FN320516 | Hypothetical protein | SEA-domain of MUC16 [PDB: 1IVZ] | 94.2 |
|  | FN320515 | Hypothetical protein | SEA-domain of TMPRSS2 [PDB: 2E7V] | 95.8 |
|  | FN320513 | Hypothetical protein | SEA-domain of TMPRSS2 [PDB: 2E7V] | 91.3 |
| Sja.14561 (3988289) | FN327139 | Egg protein CP3842 | SEA-domain of TMPRSS2 [PDB: 2E7V] | 95.6 |
|  | FN323795 | Egg protein CP3842 | SEA-domain of TMPRSS2 [PDB: 2E7V] | 95.6 |
|  | FN323796 | Egg protein CP3842 | SEA-domain of TMPRSS2 [PDB: 2E7V] | 95.6 |
|  | FN323775 | Egg protein CP3842 | SEA-domain of TMPRSS2 [PDB: 2E7V] | 95.6 |
| Sja.11325 (2672175) | AY813755 | Egg protein CP3842 | SEA-domain of TMPRSS2 [PDB: 2E7V] | 95.9 |
|  | FN320057 | Hypothetical protein | SEA-domain of TMPRSS2 [PDB: 2E7V] | 95.9 |
|  | FN320056 | Hypothetical protein | SEA-domain of TMPRSS2 [PDB: 2E7V] | 95.8 |
|  | FN320514 | Hypothetical protein | SEA-domain of TMPRSS2 [PDB: 2E7V] | 96 |
|  | FN329566^NC^ | Non-coding | N/A | N/A |
|  | BU768160^EST^ | Unknown | N/A | N/A |
|  | BU774105^EST^ | Unknown | N/A | N/A |
|  | BU770186^EST^ | Unknown | N/A | N/A |
|  | BU779051^est^ | Unknown | N/A | N/A |
| Sja.9771 (2493712) | AY570756^SST^ | Egg protein SjCP400 | SEA-domain of TMPRSS2 [PDB: 2E7V] | 92.4 |
|  | FN327121 | Somula protein | SEA-domain of TMPRSS2 [PDB: 2E7V] | 96.5 |
|  | FN327254 | Somula protein | SEA-domain of TMPRSS2 [PDB: 2E7V] | 96.5 |
|  | FN327253 | Somula protein | SEA-domain of TMPRSS2 [PDB: 2E7V] | 96.5 |
|  | FN327241 | Somula protein | SEA-domain of TMPRSS2 [PDB: 2E7V] | 96.5 |
|  | FN327233 | Somula protein | SEA-domain of TMPRSS2 [PDB: 2E7V] | 96.5 |
|  | FN327229 | Somula protein | SEA-domain of TMPRSS2 [PDB: 2E7V] | 96.5 |
|  | FN327224 | Somula protein | SEA-domain of TMPRSS2 [PDB: 2E7V] | 96.5 |
|  | FN327222 | Somula protein | SEA-domain of TMPRSS2 [PDB: 2E7V] | 96.5 |
|  | FN327216 | Somula protein | SEA-domain of TMPRSS2 [PDB: 2E7V] | 96.5 |
|  | FN327196 | Somula protein | SEA-domain of TMPRSS2 [PDB: 2E7V] | 96.5 |
|  | FN327185 | Somula protein | SEA-domain of TMPRSS2 [PDB: 2E7V] | 96.5 |
|  | FN327163 | Somula protein | SEA-domain of TMPRSS2 [PDB: 2E7V] | 96.5 |
|  | FN327158 | Somula protein | SEA-domain of TMPRSS2 [PDB: 2E7V] | 96.5 |
|  | FN327154 | Somula protein | SEA-domain of TMPRSS2 [PDB: 2E7V] | 96.5 |
|  | FN327129 | Somula protein | SEA-domain of TMPRSS2 [PDB: 2E7V] | 96.5 |
|  | FN327125 | Somula protein | SEA-domain of TMPRSS2 [PDB: 2E7V] | 96.5 |
|  | FN327115 | Somula protein | SEA-domain of TMPRSS2 [PDB: 2E7V] | 96.5 |
|  | FN327089 | Somula protein | SEA-domain of TMPRSS2 [PDB: 2E7V] | 96.5 |
|  | FN327083 | Somula protein | SEA-domain of TMPRSS2 [PDB: 2E7V] | 96.4 |
|  | FN327073 | Somula protein | SEA-domain of TMPRSS2 [PDB: 2E7V] | 96.5 |
|  | FN327057 | Somula protein | SEA-domain of TMPRSS2 [PDB: 2E7V] | 96.2 |
|  | FN327050 | Somula protein | SEA-domain of TMPRSS2 [PDB: 2E7V] | 96.5 |
|  | FN327049 | Somula protein | SEA-domain of TMPRSS2 [PDB: 2E7V] | 96.5 |
|  | FN327045 | Somula protein | SEA-domain of TMPRSS2 [PDB: 2E7V] | 96.5 |
|  | FN327042 | Somula protein | SEA-domain of TMPRSS2 [PDB: 2E7V] | 96.5 |
|  | FN327035 | Somula protein | SEA-domain of TMPRSS2 [PDB: 2E7V] | 96.5 |
|  | FN327022 | Somula protein | SEA-domain of TMPRSS2 [PDB: 2E7V] | 96.5 |
|  | FN327018 | Somula protein | SEA-domain of TMPRSS2 [PDB: 2E7V] | 96.5 |
|  | FN327014 | Somula protein | SEA-domain of TMPRSS2 [PDB: 2E7V] | 96.2 |
|  | FN327000 | Somula protein | SEA-domain of TMPRSS2 [PDB: 2E7V] | 96.5 |
|  | FN326998 | Somula protein | SEA-domain of TMPRSS2 [PDB: 2E7V] | 96.5 |
|  | FN326978 | Somula protein | SEA-domain of TMPRSS2 [PDB: 2E7V] | 96.5 |
|  | FN326973 | Somula protein | SEA-domain of TMPRSS2 [PDB: 2E7V] | 96.5 |
|  | FN326961 | Somula protein | SEA-domain of TMPRSS2 [PDB: 2E7V] | 96.5 |
|  | FN326960 | Somula protein | SEA-domain of TMPRSS2 [PDB: 2E7V] | 96.5 |
|  | FN326959 | Somula protein | SEA-domain of TMPRSS2 [PDB: 2E7V] | 96.5 |
|  | FN326930 | Somula protein | SEA-domain of TMPRSS2 [PDB: 2E7V] | 96.5 |
|  | FN326905 | Somula protein | SEA-domain of TMPRSS2 [PDB: 2E7V] | 96.5 |
|  | FN326883 | Somula protein | SEA-domain of TMPRSS2 [PDB: 2E7V] | 96.5 |
|  | FN326882 | Somula protein | SEA-domain of TMPRSS2 [PDB: 2E7V] | 96.5 |
|  | FN326881 | Somula protein | SEA-domain of TMPRSS2 [PDB: 2E7V] | 96.5 |
|  | FN326859 | Somula protein | SEA-domain of TMPRSS2 [PDB: 2E7V] | 96.5 |
|  | FN326857 | Somula protein | SEA-domain of TMPRSS2 [PDB: 2E7V] | 96.5 |
|  | FN326852 | Somula protein | SEA-domain of TMPRSS2 [PDB: 2E7V] | 96.4 |
|  | FN326851 | Somula protein | SEA-domain of TMPRSS2 [PDB: 2E7V] | 96.5 |
|  | FN326841 | Somula protein | SEA-domain of TMPRSS2 [PDB: 2E7V] | 96.5 |
|  | FN326831 | Somula protein | SEA-domain of TMPRSS2 [PDB: 2E7V] | 96.5 |
|  | FN326829 | Somula protein | SEA-domain of TMPRSS2 [PDB: 2E7V] | 96.5 |
|  | FN326822 | Somula protein | SEA-domain of TMPRSS2 [PDB: 2E7V] | 96.5 |
|  | FN326808 | Somula protein | SEA-domain of TMPRSS2 [PDB: 2E7V] | 96.5 |
|  | FN326801 | Somula protein | SEA-domain of TMPRSS2 [PDB: 2E7V] | 96.5 |
|  | FN326790 | Somula protein | SEA-domain of TMPRSS2 [PDB: 2E7V] | 96.6 |
|  | FN326770 | Somula protein | SEA-domain of TMPRSS2 [PDB: 2E7V] | 96.5 |
|  | FN326740 | Somula protein | SEA-domain of TMPRSS2 [PDB: 2E7V] | 96.5 |
|  | FN330540^NC^ | Non-coding | N/A | N/A |
| Sja.1628 (1476264) | AY570742^SST^ | Egg protein SjCP1531 | SEA-domain of TMPRSS2 [PDB: 2E7V] | 78.2 |
|  | FN320556 | Egg protein CP1531 | SEA-domain of MUC16 [PDB: 1IVZ] | 92.8 |
|  | FN320555 | Egg protein CP1531 | SEA-domain of TMPRSS2 [PDB: 2E7V] | 86.5 |
|  | FN320553 | Hypothetical protein | SEA-domain of TMPRSS2 [PDB: 2E7V] | 88.5 |
|  | FN320552 | Hypothetical protein | SEA-domain of TMPRSS2 [PDB: 2E7V] | 88.5 |
|  | FN320551 | Egg protein CP1531 | SEA-domain of TMPRSS2 [PDB: 2E7V] | 87.2 |
|  | FN320550 | Egg protein CP1531 | SEA-domain of TMPRSS2 [PDB: 2E7V] | 88.2 |
|  | FN320549 | Egg protein CP1531 | SEA-domain of TMPRSS2 [PDB: 2E7V] | 88.2 |
| Sja.2070 (1476805) | AY599749^SST^ | Egg protein CP1731 | Photosyst. II reaction protein [PDB: 3A0H] | 49.1 |
| Sja.15108 (5233833) | AY810465 | Hypothetical protein | Platelet growth factor recept. [PDB: 216W] | 18.4 |
|  | FN321062 | Hypothetical protein | SEA-domain of TMPRSS2 [PDB: 2E7V] | 93.6 |
| Sja.11840 (2895838) | FN327242 |  | SEA-domain of TMPRSS2 [PDB: 2E7V] | 96.5 |
|  | FN327131 | Hypothetical protein | SEA-domain of TMPRSS2 [PDB: 2E7V] | 96.5 |
|  | FN327087 | Hypothetical protein | SEA-domain of TMPRSS2 [PDB: 2E7V] | 96.5 |
|  | FN326854 | Hypothetical protein | SEA-domain of TMPRSS2 [PDB: 2E7V] | 96.5 |
|  | BU776301^EST^ | Unknown | N/A | N/A |
| Sja.15036 (5233761) | FN326786 | Hypothetical protein | SEA-domain of TMPRSS2 [PDB: 2E7V] | 92.5 |
|  | FN318043 | Hypothetical protein | SEA-domain of TMPRSS2 [PDB: 2E7V] | 92.5 |
|  | CX861530^EST^ | Unknown | N/A | N/A |
| Sja.1611 (1476247) | FN317637 | Hypothetical protein | SEA-domain of TMPRSS2 [PDB: 2E7V] | 93.8 |
|  | BU772954^EST^ | Unknown | N/A | N/A |
| Sja.5326 (2034920) | FN326953 | Hypothetical protein | SEA-domain of TMPRSS2 [PDB: 2E7V] | 96.5 |
|  | FN330298^NC^ | Non-coding | N/A | N/A |
| Sja.14941 (3988669) | FN320554 | Hypothetical protein | SEA-domain of TMPRSS2 [PDB: 2E7V] | 93.6 |
| Sja.14627 (3988355) | FN319007 | Hypothetical protein | SEA-domain of TMPRSS2 [PDB: 2E7V] | 94.1 |
| Sja.14614 (3988342) | FN320058 | Hypothetical protein | SEA-domain of TMPRSS2 [PDB: 2E7V] | 95.6 |
| Sja.13298 (3987026) | FN320059 | Hypothetical protein | SEA-domain of TMPRSS2 [PDB: 2E7V] | 88.5 |
| Sja.13882 (3987610) | FN330716^NC^ | Non-coding | N/A | N/A |
| Sja.14071 (3987799) | FN329677^NC^ | Non-coding | N/A | N/A |
| Sja.13956 (3987684) | FN330422^NC^ | Non-coding | N/A | N/A |
| Sja.14095 (3987823) | FN329269^NC^ | Non-coding | N/A | N/A |

* This is an indication of the confidence level and precision of the prediction, expressed in percentage. (**^SST^**):The original signal sequence trap identified transcripts. (**^EST^**) and (**^NC^**) are expressed sequence tags (ESTs) and non-coding transcripts respectively. Structural modeling was not performed for ESTs and non-coding genes (N/A).
