# Supplementary material for: Characterization of a Gene Family Encoding SEA (Sea-urchin Sperm Protein, Enterokinase and Agrin)-Domain Proteins with Lectin-Like and Heme-Binding Properties from Schistosoma japonicum
Source: PLoS Negl Trop Dis. 2014 Jan 9;8(1):e2644. doi: 10.1371/journal.pntd.0002644 (PMC3886910; doi:10.1371/journal.pntd.0002644)
Supplement: Table S2 — Developmental stage specific expression of Schistosoma japonicum SEA-domain containing genes. The developmental stage specific expression of the candidate genes expressed as copy number per nanogram of cDNA. The detailed data statistics of the data plotted in Figure 3 is reproduced here to show mean values and standard deviations of each candidate at each developmental stage of the parasite. (DOCX) [file pntd.0002644.s009.docx]

**Table S2. Developmental Stage Specific Expression of *Schistosoma* *japonicum* SEA-domain encoding genes**

|  | Egg | Sporocyst | Cercaria | Somula | Male | Female | Mix Adult | p-value |
| --- | --- | --- | --- | --- | --- | --- | --- | --- |
| SjCP3842 | 252±370.1 | 2474±627.2 | 2871±98.4 | 543.4±64.1 | 2000±453.9 | 4846±302.1 | 5680±370.9 | p<0.004 |
| SjCP1084 | 1737±206.6 | 4.8±0.3 | 117±14.5 | 10.5±1.6 | 2.7±0.1 | 2.8±0.1 | 15.8±0.4 | p<0.005 |
| SjCP1531 | 124.5±23.9 | 17.9±2.3 | 11.9±4.5 | 14.5±0.8 | 148.5±26.8 | 190±22.8 | 344.6±5.7 | p<0.005 |

NB: Presented statistics is Mean copy number/ng of cDNA ± standard deviation (SD)
